# Structure snapshots reveal the mechanism of a bacterial membrane lipoprotein *N*-acyltransferase

**DOI:** 10.1126/sciadv.adf5799

**Published:** 2023-06-30

**Authors:** Luke Smithers, Oksana Degtjarik, Dietmar Weichert, Chia-Ying Huang, Coilín Boland, Katherine Bowen, Abraham Oluwole, Corinne Lutomski, Carol V. Robinson, Eoin M. Scanlan, Meitian Wang, Vincent Olieric, Moran Shalev-Benami, Martin Caffrey

**Affiliations:** ^1^ School of Medicine and School of Biochemistry and Immunology, Trinity College Dublin, Dublin D02 R590, Ireland.; ^2^ Department of Chemical and Structural Biology, Weizmann Institute of Science, Rehovot 7610001, Israel.; ^3^ Swiss Light Source, Paul Scherrer Institute, CH-5232 Villigen, Switzerland.; ^4^ School of Chemistry, Trinity College Dublin, Dublin D02 R590, Ireland.; ^5^ Department of Chemistry, University of Oxford, South Parks Road, Oxford OX1 3QU, UK.

## Abstract

Bacterial lipoproteins (BLPs) decorate the surface of membranes in the cell envelope. They function in membrane assembly and stability, as enzymes, and in transport. The final enzyme in the BLP synthesis pathway is the apolipoprotein *N*-acyltransferase, Lnt, which is proposed to act by a ping-pong mechanism. Here, we use x-ray crystallography and cryo–electron microscopy to chart the structural changes undergone during the progress of the enzyme through the reaction. We identify a single active site that has evolved to bind, individually and sequentially, substrates that satisfy structural and chemical criteria to position reactive parts next to the catalytic triad for reaction. This study validates the ping-pong mechanism, explains the molecular bases for Lnt’s substrate promiscuity, and should facilitate the design of antibiotics with minimal off-target effects.

## Teaser

Structures of the bacterial lipoprotein N-acyltransferase in nine discrete conditions reveal the enzyme’s reaction mechanism.

## INTRODUCTION

Lipoproteins are ubiquitous with representatives in all domains of life. Conjugated lipids include glycosylphosphatidylinositols, prenols, cholesterol, and fatty acids (*1*, *2*). Lipidation regulates signal transduction, membrane trafficking, and apoptosis. Bacteria have specialized lipoproteins [bacterial lipoproteins (BLPs)], which act as enzymes, inhibitors, and components of complexes involved in nutrient uptake, membrane assembly, and photosynthesis (*2*, *3*). Because the lipid modification at an invariant cysteine in BLPs is a molecular motif unique to bacteria, the innate immune system has evolved Toll-like receptors (TLRs) to recognize BLPs as foreign and, upon detection, to launch protective responses (*4*–*7*). BLPs have been the focus of vaccine development and are used in nanopore sequencing (*8*–*14*).

**Fig. 1.**
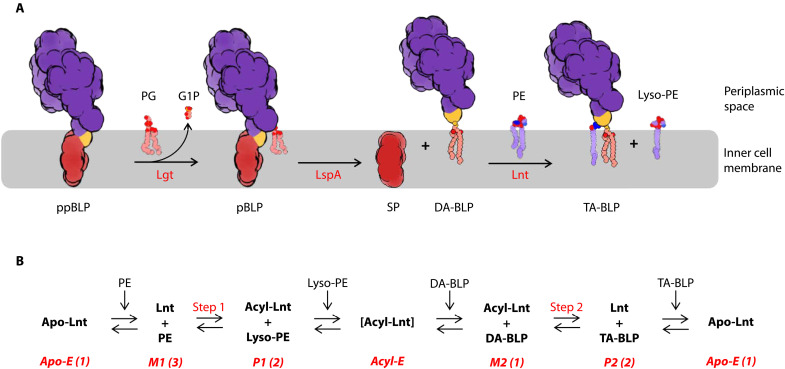
Canonical BLP processing pathway and Lnt reaction scheme. (**A**) Posttranslational modification of ΒLPs by the canonical pathway. A prepro-BLP (ppBLP) substrate is shown inserted in the plasma membrane with a purple ultradomain, a red signal peptide, and a yellow lipobox cysteine. Lgt catalyzes the transfer of diacylglyceryl from phosphatidylglycerol (PG) to the thiol of the lipobox cysteine, generating a pro-BLP (pBLP) and glycerol-1-phosphate (G1P). LspA cleaves the N-terminal signal peptide from the pBLP to form diacylglyceryl BLP (DA-BLP) and free signal peptide (SP). Lnt then transfers the *sn*-1 acyl chain from a GPL [phosphatidylethanolamine (PE) shown here] to the N-terminal amino group of DA-BLP. TA-BLP and lyso-PE are formed as products. (**B**) The proposed ping-pong mechanism of the Lnt *N*-acyl transfer reaction. A ping-pong or double-displacement reaction involves two substrates. The first substrate binds to and chemically modifies the enzyme. In the process, the altered substrate becomes the first product. It leaves the active site before the second substrate engages with the enzyme to pick up the modification, thereby becoming the second product. When the second product exits the active site, the enzyme is restored to its original condition, ready for another round of reaction. Apo-E, apo enzyme; M1/M2, first and second Michaelis complexes; P1/P2, first and second product complexes; acyl-E, acylated Lnt. The number in parentheses refers to the number of experimental structures reported in this study for each state in the reaction.

**Fig. 2.**
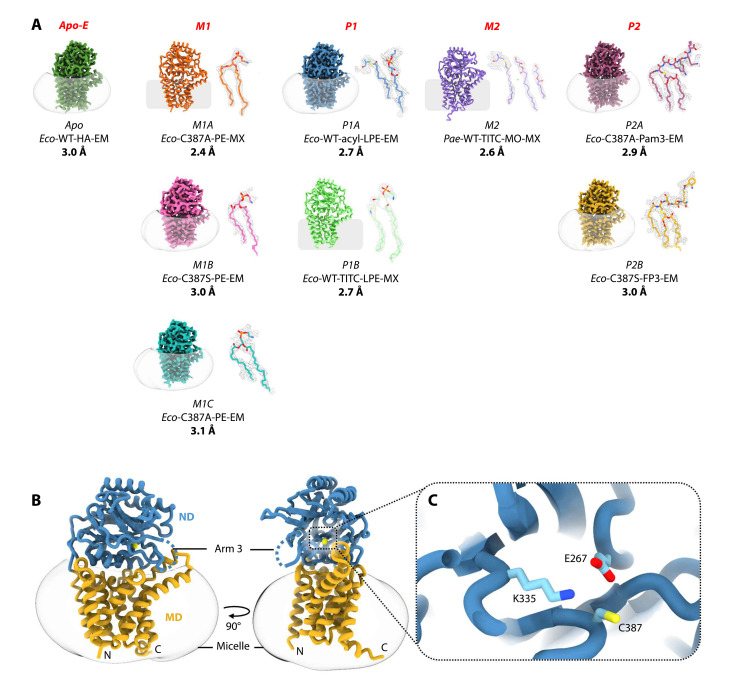
Structures solved as part of this study representing different states along the Lnt ping-pong reaction mechanism. (**A**) The nine solved structures are arranged on the basis of the state they represent (apo, M1, P1, M2, and P2) and are shown embedded in detergent micelles (cryo-EM structures) or with approximate membrane boundaries (MX structures). Bound substrates, modifications, and products are shown to the right of each structure in their corresponding electron densities (MX) or maps (EM). The descriptor provided below each structure abbreviation indicates the organism from which each is derived (*Eco*, *E. coli*; *Pae*, *P. aeruginosa*), mutations (WT, C387A, and C387S), bound substrates, products, modifications [PE, tetradecyl-1-isothiocyanate (TITC), acyl, lyso-PE (LPE), monoolein (MO), Pam3 (Pam_3_CSK_4_), and FP3], whether the structure is derived from x-ray crystallography (MX) or cryo–electron microscopy (cryo-EM), and the resolution. (**B**) Overall structure of the Apo-E form of Lnt in a detergent micelle obtained by cryo-EM. The nitrilase-like domain (ND) is shown in blue and the membrane domain (MD) in yellow. Arm 3, for which density is poor, is indicated as a dashed blue line. The cytoplasm-facing N and C termini are labeled, and the catalytic cysteine is shown as a yellow sphere. A dashed box identifies the location of the active site. (**C**) Magnified view of the Lnt active site with the catalytic triad residues—E267, K335, and C387 (*E. coli* numbering)—in stick representation.

**Fig. 3.**
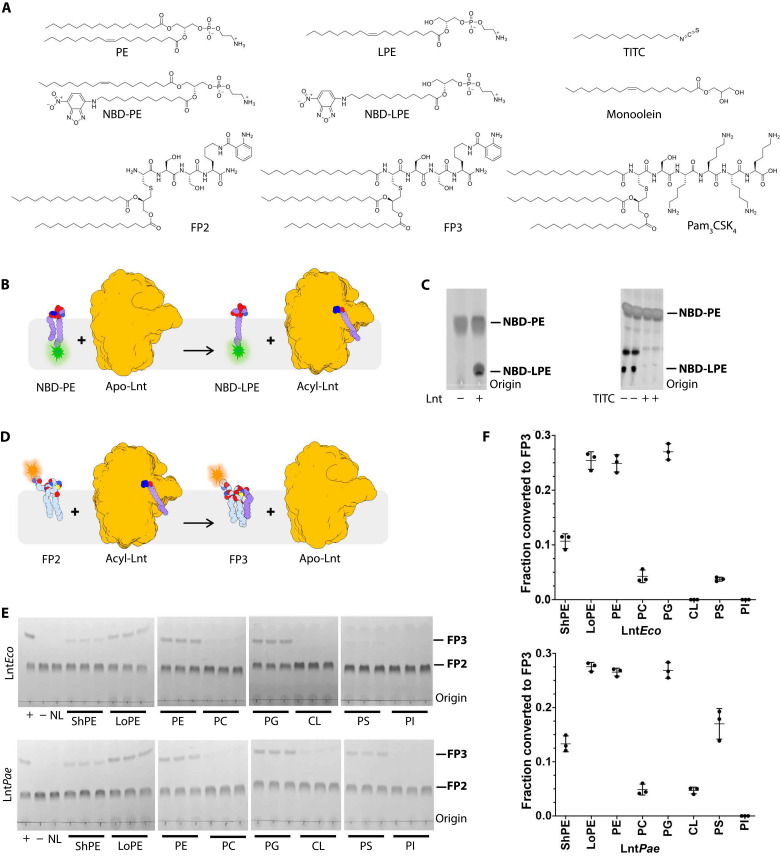
Overview of Lnt substrates, products, analogs, and assays to quantify its *N*-acyltransferase activity. (**A**) Structures of chemicals used [NBD-PE, nitrobenzoxadiazole-labeled PE; FP2, Cys(Pam_2_)-Ser-Ser-Lys(Abz)-NH_2_; Abz, 2-aminobenzoyl is a fluorophore; FP3, Cys(Pam_3_)-Ser-Ser-Lys(Abz)-NH_2_; Pam_3_CSK_4_, Cys(Pam_3_)-Ser-(Lys)_4_]. (**B**) Cartoon representation of the step 1 assay. PE, labeled at the *sn*-2 chain (NBD-PE), is incubated with Lnt. Auto S-acylation generates the fluorescent product, NBD-LPE. (**C**) Thin-layer chromatography (TLC) results obtained for the assay in (B). NBD-PE and NBD-LPE are indicated in the presence (+) and absence (−) of Lnt (left). In the right, TITC modification (+) inactivates the enzyme by producing a stable acylated Lnt, compared to the untreated enzyme (−). The difference in the position of NBD-PE and NBD-LPE on the TLC arises from a difference in polarity. (**D**) Cartoon representation of the step 2 assay. FP2 and FP3 are extracted from the reaction mix and quantified by TLC. (**E**) Step 2 assay assessing the GPL specificity of Lnt*Eco* and Lnt*Pae* using hydroxylamine (HA)–treated enzyme. Symbols below each lane indicate the GPL types tested: (+) positive control with untreated Lnt and POPE, (−) no enzyme negative control, (NL) no added lipid control, (ShPE) short PE (12:0-12:0 PE), and (LoPE) long PE (18:0-20:4 PE). All other GPLs tested [PE, PC (phosphatidylcholine), PG, CL (cardiolipin), PS (phosphatidylserine), and PI (phosphatidylinositol)] are of the palmitoyl-oleoyl form. Data are end point (90-min) measurements at 37°C. The difference in the position of FP2 and FP3 on the TLC arises from a difference in polarity. (**F**) Densitometric analysis of the total fractional conversion of FP2 to FP3 for data in (E) is reported as scatterplots that include averages (*n* = 3) and SDs. In the current study, PE and PG are equally active as substrates. In earlier work from this group, PE was the preferred lipid substrate (*37*).

The canonical pathway of BLP synthesis includes three enzymes, phosphatidylglycerol prolipoprotein diacylglyceryl transferase (Lgt), lipoprotein signal peptidase (LspA), and Lnt (Fig. 1A) that are essential for normal growth in many pathogenic bacteria (*2*). With no equivalents in humans, they are attractive potential targets for antibiotic development and have been under intense scrutiny recently (*15*–*24*). Lnt is a 57-kDa protein that is present in diderm bacteria. It converts a diacylated BLP (DA-BLP) to a triacylated BLP (TA-BLP) using glycerophospholipids (GPLs) as acyl donors. Conversion to the TA-BLP form prepares BLPs for trafficking to the outer membrane and switches the TLR system they activate (*25*–*27*). The reaction is proposed to proceed by a ping-pong mechanism (Fig. 1B) at catalytic triad residues Glu^267^, Lys^335^, and Cys^387^ (*Escherichia coli* numbering; Fig. 2C) (*28*). Lnt is promiscuous with regard to its GPL and BLP substrates (Fig. 3, A, E, and F, and fig. S1) (*28*, *29*).

The Lnt reaction is proposed to begin with the enzyme in its apo state (Fig. 1B). Upon binding a GPL, preferentially phosphatidylethanolamine (PE) or phosphatidylglycerol (PG) (Fig. 3, E and F) (*29*–*33*), it forms the first Michaelis complex, M1. Once bound, the acyl chain at the *sn*-1 position on the glyceryl of the GPL is transferred to the sulfur at the catalytic cysteine, forming the thioacylated enzyme (via a thioester linkage) and lyso-GPL as the first product. When lyso-GPL exits the active site, the first product complex, P1, converts to the acyl-enzyme intermediate, acyl-Lnt (acyl-E). The second substrate, DA-BLP, enters the active site, forming the second Michaelis complex, M2. The acyl chain at the catalytic cysteine is transferred to the N-terminal α-amino group of DA-BLP producing TA-BLP and the second product complex, P2. Upon departure of TA-BLP from the active site, the apo enzyme is restored, primed for another round of catalysis.

Several crystal structures of Lnt are known, all are of the apo enzyme (*34*–*38*). Thus, the molecular details of how substrates, intermediates, and products engage and transform at and around the catalytic center remain obscure. Likewise, the chemical and physical features of the binding pocket that provide the proximity and orientation necessary for efficient catalysis have not, until now, been structurally characterized. In this study, we combined macromolecular x-ray crystallography (MX) and single-particle cryo–electron microscopy (cryo-EM) to record snapshots of discrete states through the *N*-acyltransferase reaction. We obtained nine structures within the resolution range of 2.4 to 3.1 Å, capturing the enzyme in its apo and its Michaelis and product complex states (Figs. 1B and 2A). The structures explain how Lnt engages with and transforms two substrates as well as produces and releases two products by a double-displacement mechanism. The enzyme has been characterized biochemically using activity assays and mass spectrometry (MS). Together, this study provides a structural delineation of the entire enzymatic pathway, revealing the catalytic mechanism of an enzyme of great physiological and potential therapeutic value.

## RESULTS

### Enzyme production, characterization, and structure determination

The goal of this study was to record three-dimensional (3D) structure snapshots to define the conformational continuum as Lnt progresses through the proposed six states of its *N*-acylation reaction. Recombinant Lnt from *E. coli* (Lnt*Eco*) and *Pseudomonas aeruginosa* (Lnt*Pae*) were produced for analysis in detergent micelles. The enzyme was captured in its assorted states in two ways. The first involved using mutants, where the catalytic cysteine was replaced by either alanine (inactive) or serine (low activity) (fig. S2) (*35*). The second approach used a covalent modification at the catalytic cysteine [tetradecyl-1-isothiocyanate (TITC); Fig. 3, A and C, and fig. S1]. Functional characterization was performed by thin-layer chromatography (TLC) and native MS. The assay to monitor the first reaction step used a nitrobenzoxadiazole (NBD)–labeled PE (NBD-PE) substrate, where the label is at the terminus of the *sn*-2 chain (Fig. 3A). When the unlabeled *sn-*1 chain is transferred to the catalytic cysteine, NBD-labeled lyso-PE (NBD-LPE) is produced and quantified (Fig. 3, B and C). Step 2, in which the acyl chain on Lnt is transferred to the N-terminal cysteine of DA-BLP, was monitored using a fluorescent lipopeptide substrate, Cys(Pam_2_)-Ser-Ser-Lys(Abz)-NH_2_ (FP2) (Fig. 3, D and E). FP2 has a dipalmitin in thioether linkage to the N-terminal cysteine. The fluorophore, 2-aminobenzoyl (Abz), is attached to the ε-amino group of the C-terminal lysine. The product of the reaction, Cys(Pam_3_)-Ser-Ser-Lys(Abz)-NH_2_ (FP3) (Fig. 3A), was monitored by TLC and MS (fig. S3).

Structure determination was performed by MX and cryo-EM. For MX, we used the in meso (lipid cubic phase) and in surfo (vapor diffusion) methods for crystallization. Three crystal structures were solved with resolutions from 2.4 to 2.7 Å (Fig. 2A, fig. S4, and table S1). For cryo-EM, the protein, in a detergent micelle, yielded six map reconstructions with resolution values ranging from 2.7 to 3.1 Å (Fig. 2A, figs. S5 and S6, and table S2). Commercial or in-house synthetized BLP peptide analogs (BLPtide), shown to be effective substrates and products, were used to capture the different states in the reaction (Fig. 3A).

### Overall Lnt architecture

The nine structures determined in this study recapitulate Lnt in distinct states through the *N*-transacylase reaction. Despite the disparate conformations of the binding pocket and its surroundings, the overall architecture of the enzyme is similar between states. It will be introduced now based on the structure of the apo enzyme (Fig. 2B).

Lnt is composed of two equally sized domains, a membrane domain (MD) and a nitrilase-like domain (ND) (Fig. 2B). The MD has eight helices (H1 to H8) that cross the membrane with both N and C termini in the cytoplasm. The globular ND sits atop the MD and contains an αββα sandwich fold. While the ND core is conformationally stable, the enzyme as a whole has nine loops at its front end that appear highly mobile (arms 1 to 9; figs. S6, B and D; S7A; and S8B). The catalytic cysteine resides in a loop connecting the two lower α and β layers of the ND (fig. S7B). It passes between arms 1 and 9 on the right-hand side (facing into the catalytic center) and arm 3 on the left (fig. S7A). Arm 1 is made of the periplasmic extensions of H3 and H4 and a loop connecting them in the MD. The loop between β8 and α4 at the bottom half of the ND sandwich constitutes arm 9. Arm 3 is a 40-residue-long loop between β strands linking the two halves of the αββα sandwich. The bottom of the binding pocket is created by a linker, L1, connecting H5 and H6. Collectively, these arms and the base of the pocket create a hydrophobic channel that accommodates the acyl chains of the substrates and products in their passage between the membrane and the catalytic center.

### Apo state

The apo state corresponds to Lnt with its catalytic site free of substrate, product, and thioacylation. In the cell membrane, the enzyme autoacylates, where the dominant form is considered to be thioacylated (*35*, *39*). Each Lnt co-purifies with approximately 20 GPLs that derive from the cell membrane (fig. S9A). These can act as substrates for Lnt (*34*). Nonetheless, all Lnt structures determined to date provide no evidence of acylation. They show either an empty binding pocket for structures obtained by the in surfo method (*34*, *38*) or a pocket with monoolein molecules that derive from the crystallization medium when crystals are grown by the in meso method (*35*–*38*). Presumably, the lack of thioacylation is due to the labile nature of the thioester linkage, which, over the course of a crystallization trial, is prone to hydrolysis. Cryo-EM does not involve protracted incubations, and to capture the apo state by this method, the enzyme must first be freed of thioacylation and adventitious phospholipids. A hydroxylamine (HA) treatment was implemented for this purpose (fig. S9B) (*39*).

The HA-treated protein was enzymatically active only in the presence of added GPLs, indicating that adventitious phospholipids had been hydrolyzed (Fig. 3E). Cryo-EM analysis provided a 3.0-Å reconstruction that had an active site free of thioacylation, of all lipid, and of BLP substrates and products (Apo; Fig. 2A and figs. S5 and S6). Comparison of the apo reconstruction with the previously determined structures of Lnt obtained by MX revealed a highly similar architecture with average root mean square deviation (RMSD) values for Cα atoms of 0.92 Å (fig. S8A). The cryo-EM structure has 16 residues (Glu^348^ to Ser^363^) in arm 3 that have no density. Similarly, in earlier MX structures, arm 3 has missing residues or residues characterized by high B factor values that indicate motion and disorder (fig. S8B) (*34*, *35*, *37*, *38*). These findings suggest that arm 3 is flexible and, given its location in Lnt, we propose that it is involved in substrate and product binding. Parts of arm 3 are disordered to varying degrees in our substrate- and product-bound structures that recapitulate different states in the catalytic pathway (vide infra) (figs. S4 and S6D). This supports our hypothesis that arm 3 is crucial for engaging with substrates and products. The mobility of arm 3 in the apo state also likely primes the enzyme to bind its first substrate, GPL.

### First Michaelis complex: M1 state

**Fig. 4.**
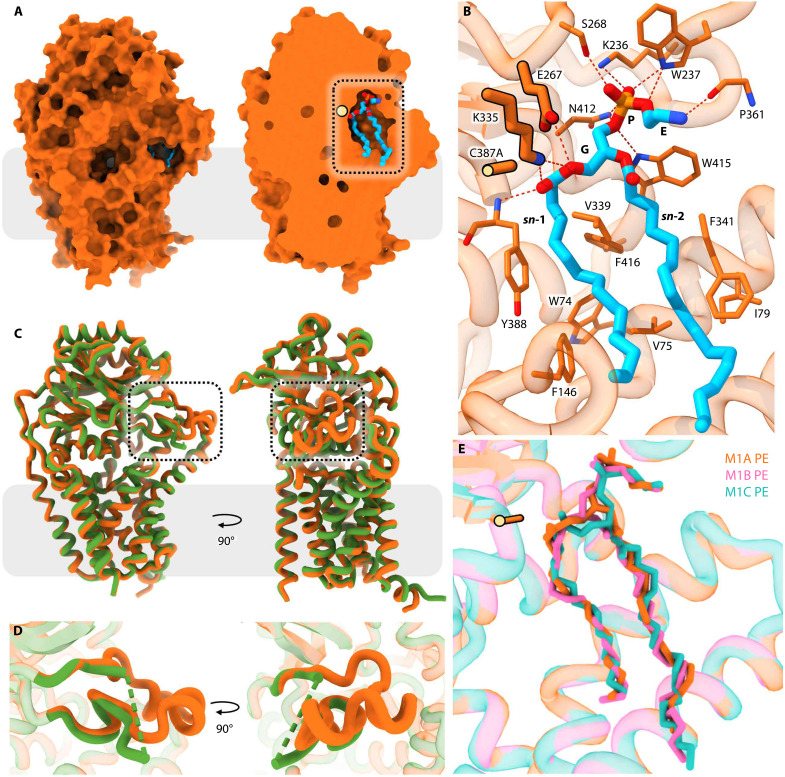
Structure of Lnt in complex with PE representing the first Michaelis complex. (**A**) Overall structure in surface representation of Lnt*Eco* bound to PE (M1A). Lnt is colored orange. PE is colored by element with cyan carbons. A surface representation is shown on the left. A clipped version, on the right, reveals the active site, with Cys387Ala shown as a light yellow sphere. The dashed box identifies the region magnified in (B). (**B**) Interactions between residues in Lnt*Eco* Cys387Ala and PE in M1. Red dashed lines identify hydrogen bonding partners. Other residues form hydrophobic interactions. E (ethanolamine), P (phosphate), and G (glycerol) correspond to moieties in the PE headgroup. *sn*-1 and *sn*-2 are the acyl chains of PE. The bond linking the Cα and methyl side chain of the Cys387Ala residue is highlighted with a black outline and a yellow sphere. (**C**) Alignment of the apo (green) and M1A (orange) structures (RMSD of 0.71 Å over Cα). This RMSD value is artificially low because arm 3 is absent in the apo structure. A dashed box indicates the region around arm 3 magnified in (D). (**D**) Comparison of arm 3 in the apo (green) and M1A (orange) structures. The dashed line demarks the portion of arm 3 in apo that is not in well-defined electron density. The missing residues extend from Glu^348^ to Ser^363^ inclusive. (**E**) View into the active site where the three M1 representative structures (M1A in orange, M1B in pink, and M1C in teal) have been aligned. Cys387Ala is highlighted as described in (B).

The M1 state has Lnt in complex with its GPL substrate (Fig. 1B). Our original intent was to capture a structure of M1 crystallographically, and the in meso crystallization method was considered for that purpose. The in meso method uses the cubic mesophase, composed of monoolein (Fig. 3A), as the medium in which crystals grow. Because monoolein is present at a concentration of ~2 M, it can outcompete substrates and products. We, therefore, resorted to in surfo crystallization (movie S1) in the presence of added *E. coli* GPLs that include PE, PG, and cardiolipin (CL). Because PE and PG are substrates, we used the inactive variant, Cys387Ala, to capture a representation of the M1 state (M1A; Fig. 2A). The corresponding structure was obtained at a resolution of 2.4 Å. The ester at the *sn*-1 acyl chain of PE was positioned in the binding pocket next to Cys387Ala for reaction (Fig. 4, A and B). This structure was identical to cryo-EM reconstructions of the Cys387Ser and Cys387Ala mutant forms captured in complex with PE (M1B and M1C) at resolutions of 3.0 and 3.1 Å, respectively (Figs. 2A and 4E). The main difference between the two sets was that the MX structure was obtained with added PE, while the PE observed by cryo-EM was derived from the cell. Because the three structures are essentially identical, all are viewed as M1 representatives. Because the MX structure has the highest resolution, it will be used to describe the M1 state.

The overall structure of M1 is remarkably similar to that of apo Lnt (RMSD, 0.71 Å over Cα atoms; Fig. 4C). The largest differences show up in arm 3, which, in the apo form, is partly disordered and, in M1, is stably folded over the PE molecule (Fig. 4D). The PE is bound with its glyceryl phosphoethanolamine headgroup in front of the catalytic triad and its acyl chains extending along the hydrophobic groove that acts as a conduit between the membrane and the active site (Fig. 4, A and B). As a result, except for the acyl chain termini, the PE molecule is completely buried in the enzyme (Fig. 4A). This suggests that PE enters from the membrane via the hydrophobic portal between arms 1 and 3 with the acyl chains pointing down and the polar headgroup in the periplasm. For productive engagement, PE must enter oriented with the acyl chains one behind the other and the *sn*-1 chain on point. This ensures passage along the narrow corral with the ester bond at the *sn*-1 position proximal to the catalytic cysteine. The polar headgroup of PE is arranged with the glyceryl and phosphate extended toward the top of the binding cavity. At the phosphate, the ethanolamine has its long axis oriented toward the front of the enzyme. The two acyl chains of PE are in the narrow hydrophobic groove at the base of the binding pocket with the *sn*-1 chain running along the back and the *sn*-2 chain to the front. The chains adopt a somewhat unexpected orientation being tilted at an angle of ~45° with respect to the membrane plane (Fig. 4A). Our interpretation is that this tilting is a necessary part of a productive substrate engagement with the enzyme, which positions the *sn*-1 ester bond next to Cys^387^ for reaction.

Interactions between the enzyme and PE are multifarious, with the bulk involving conserved residues. The M1 state structure therefore provides a rationalization of the results of studies to explore the role played by different residues in the structure and function of the enzyme (Fig. 4). Thus, residues at the catalytic center are suitably poised next to the *sn*-1 ester bond and the headgroup in PE to facilitate thioacylation followed by acyl transfer. The phosphate in the headgroup is held in the binding pocket by coordination with several conserved amino acids, while the acyl chains make contact with critical apolar residues (Fig. 4B and fig. S10). The ethanolamine is less extensively coordinated with just one hydrogen bond to Pro^361^ in arm 3. This weak interaction helps explain the promiscuity of Lnt regarding the GPL substrate type.

GPL specificity was investigated using an HA-treated enzyme to avoid background N-acylation from adventitious GPLs (fig. S9B). Of the potential substrates tested, palmitoyloleoylphosphatidylglycerol (POPG) and palmitoyloleoylphosphatidylethanolamine (POPE) were effective acyl donors for Lnt*Eco* and Lnt*Pae*. Long (18:0-20:4)– and short (12:0-12:0)–chained PEs showed contrasting activities, with short PE, by far, the weaker substrate. These findings are consistent with the acyl chain binding region of Lnt, which is open-ended and is able to accommodate extra-long chains. Short PEs presumably make less-favorable contacts with the apolar pocket and are a weaker substrate as a result. Phosphatidylinositol (PI), phosphatidylcholine (PC), and CL showed little or no activity with Lnt (Fig. 3, E and F, and fig. S9).

### Acyl-enzyme–lipid product complex: P1 state

The P1 state has Lnt thioacylated at the catalytic cysteine and bound to the lipid product, lyso-PE (Figs. 1B and 2A). Given the labile nature of the thioester linkage, we produced a chemically stable variant where the catalytic cysteine is linked to an alkyl chain via a dithiocarbamate upon reaction with TITC (Fig. 3A). Crystals of the TITC-modified protein (Lnt-TITC), grown in the presence of *E. coli* GPLs, diffracted to 2.66 Å. Unexpectedly, the corresponding structure had Lnt-TITC in complex with lyso-PE (P1B; Fig. 2A and table S1). The lysolipid presumably originated from the *E. coli* GPL added to facilitate crystallogenesis. In a parallel cryo-EM study, we obtained a 2.7-Å reconstruction of wild-type (WT) Lnt*Eco* bound to lyso-PE (P1A; Fig. 2A). A long lobe of density is seen extending from the catalytic cysteine that we interpret as corresponding to the naturally acylated form of Lnt (Fig. 2A and fig. S6C). Additional density, into which lyso-PE was modeled, is seen next to the acyl chain at Cys^387^. The two elongated lobes are distinct, and the modification is taken to represent P1. The lyso-PE had not been added during sample preparation. Rather, it likely formed in situ from adventitious GPLs. In the absence of a second substrate, the enzyme presumably autoacylated using passenger GPL, and the product, lyso-PE, remained in the pocket. This is consistent with native MS showing signatures of Lnt covalently modified with palmitate (fig. S3, A and B). Despite the very different biochemical approaches, the two P1 structures obtained are essentially identical (figs. S11 and S12). Because the cryo-EM structure is of the native enzyme with the P1 state produced in situ, the rest of the discussion will focus primarily on it.

**Fig. 5.**
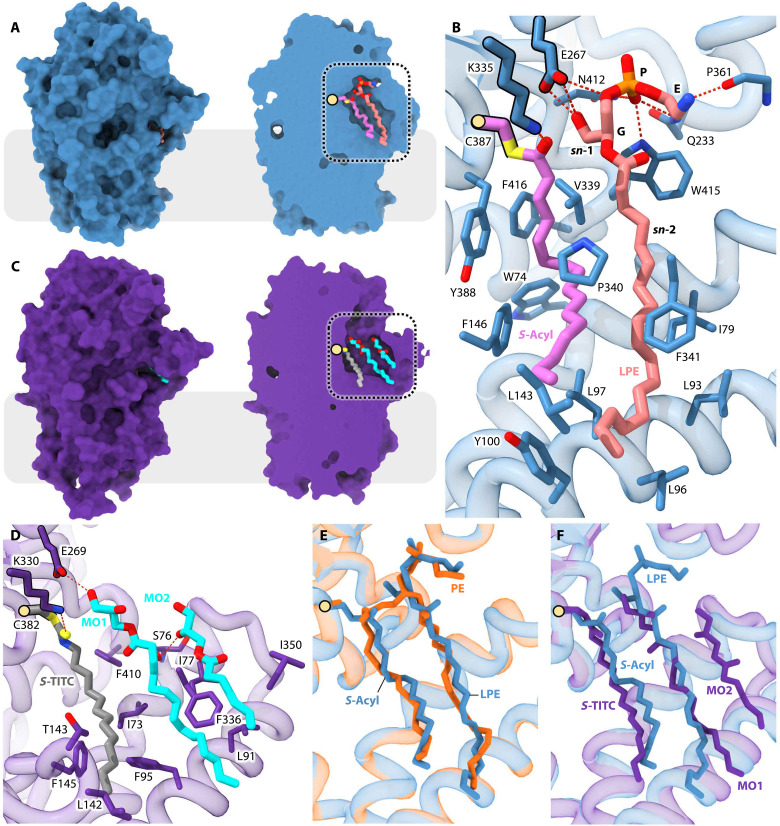
Structures of acyl-Lnt in complex with LPE (P1 state) and of TITC-modified Lnt in complex with monoolein (M2 state mimic). (**A**) Overall structure in surface representation of acyl-Lnt*Eco* bound to LPE (P1A). Lnt is colored blue, while LPE and the *S*-acyl chain are colored by element with salmon and pink carbon atoms, respectively. A surface representation is shown on the left and a clipped version on the right reveals the active site. Cys^387^ is shown as a yellow sphere, and a dashed black box indicates the region magnified in (B). (**B**) Interactions between residues in Lnt*Eco* and the *S*-acyl chain and LPE. Red dashed lines identify hydrogen bonding partners. Other residues form hydrophobic interactions. E (ethanolamine), P (phosphate), and G (glycerol) correspond to moieties in the LPE headgroup. *sn*-2 is the acyl chain of LPE. (**C**) Overall structure in surface representation of TITC-Lnt*Pae* bound to two monoolein (MO) molecules (M2 mimic). Lnt is colored purple, while MO and the TITC modification are colored by element with cyan and gray carbon atoms, respectively. A surface representation is shown on the left and a clipped version on the right shows the active site. A dashed box indicates the region magnified in (D). (**D**) Interactions between residues in Lnt*Pae* and TITC, as well as MO1 and MO2. Red dashed lines identify hydrogen bonding partners. Other residues form hydrophobic interactions. (**E**) View into the active site of an alignment between M1A (orange) and P1A (blue). (**F**) View into the active site of an alignment between the P1A (blue) and the M2 mimetic (purple).

The overall architecture of Lnt in the P1 state resembles that of M1. The two differ in the identity of bound lipids. In M1, the lipid is PE. In P1, bound lipid includes the chain linked to Cys^387^ and lyso-PE. Both sets of lipids sit in similar locations in the binding pocket (Fig. 5E). In P1, as observed in the M1 state, the lipids are buried within the protein, except for the chain termini that extend into the membrane (Fig. 5A). In contrast to the *S*-acyl chain, which is directly at Cys^387^, in the P1 state, the acyl chain of lyso-PE is shifted away from the catalytic center toward the front of the enzyme. However, it occupies the same volume and is superimposable on the *sn*-2 chain of PE in M1 (Fig. 5E). The polar headgroup, which includes the ester, glyceryl, phosphate, and ethanolamine, likewise has shifted away from the catalytic center compared to its counterpart PE in M1. In addition, there are small differences that likely contribute to the P1 state, favoring the release of lyso-PE. An examination of the contacts between the enzyme and the lipids shows that interactions are fewer in the case of lyso-PE (Fig. 5B). These center on the headgroup, phosphate in particular. As a result of acyl transfer from PE in the first reaction step, a hydroxyl is generated at the *sn*-1 position on the glyceryl of lyso-PE. This increases its aqueous solubility that favors partitioning out of the binding pocket, possibly into the periplasm. The changes documented regarding the relatively weak interaction between the lyso-PE and the enzyme are consistent with this proposal. Furthermore, the end of arm 3 is less structured in P1 compared to M1 (fig. S6D). This is interpreted as preparing the enzyme for release of lyso-PE, engagement with the second substrate, and advancement to the next step in the reaction.

### Acylated enzyme intermediate: Acyl-Lnt state

To capture the acyl-enzyme intermediate in isolation, it is necessary for the first product of the reaction, lyso-PE, to have departed and for the DA-BLP substrate not to have entered the active site (Fig. 1B). It is unclear that such an intermediate forms as an isolated entity during reaction because the thioester is inherently labile. Nonetheless, in earlier work from the group, the acyl-enzyme intermediate state was generated in silico by bonding a palmitoyl group to the catalytic cysteine of apo Lnt and subjecting it to molecular dynamics simulations (*37*). In the current study, we sought to generate a structure representative of the acyl-enzyme intermediate using Lnt-TITC as a mimic. Trials were set up with this construct using the in meso and in surfo methods. The in surfo method provided a structure of TITC-modified Lnt*Eco* with lyso-PE bound reminiscent of the P1 state (P1B; Fig. 2A). By the in meso method, a structure of the TITC-modified Lnt*Pae* was obtained with two monooleins in the binding pocket next to the TITC-modified cysteine (M2; Fig. 2A). We consider this a better mimetic of the second Michaelis complex, M2, than the acyl-enzyme intermediate, as discussed in the following section.

### Second Michaelis complex: M2 state

The M2 state has the second substrate, DA-BLP, in the binding pocket next to the thioacylated cysteine of Lnt ready for step 2. In an attempt to generate a structure representative of M2 by cryo-EM, we initially used the Cys387Ser mutant with the DA-BLP analog, FP2. A 3.0-Å resolution reconstruction was obtained, but in the final map, we found the TA-BLPtide product, FP3, indicating that *N*-acylation had taken place and that the P2 state had been generated (P2B; Fig. 2A). Thus, the M2 state could not be captured as proposed. In parallel, in meso co-crystallization trials were set up with TITC-labeled Lnt from *P. aeruginosa* and FP2, which yielded a structure to 2.6 Å (M2; Fig. 2A). In this case, the alkyl chain at the catalytic cysteine (Cys^382^ in Lnt*Pae*) was in density, but FP2 was not found. Instead, two monooleins from the mesophase were observed in the pocket (Fig. 5, C and D). We refer to this structure as the M2 mimic where the monooleins are taken as surrogates for the acyl chains in the DA-BLP substrate. Note that the structure of Lnt*Eco* and Lnt*Pae* are remarkably similar with RMSD values over 501 residues of 1.2 Å and catalytic triads that superpose almost exactly (*37*).

**Fig. 6.**
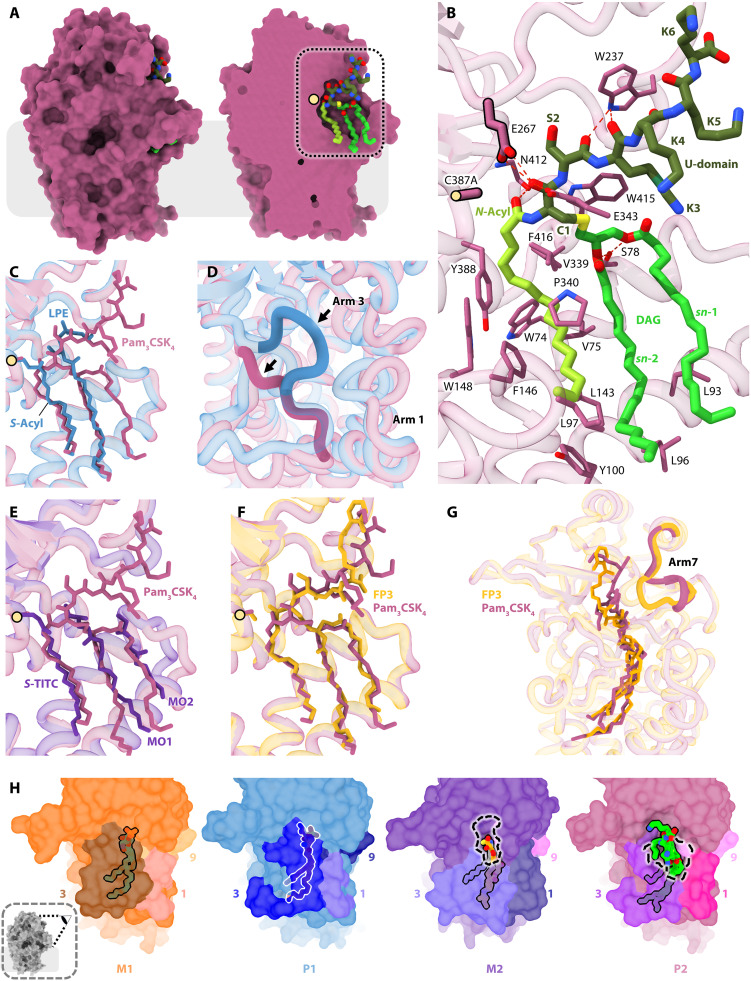
Structure of Lnt in complex with TA-BLPtide products (P2 state). (**A**) Overall structure of Lnt*Eco* bound to Pam_3_CSK_4_ (P2A). Lnt is colored light purple, while P2A is colored by element with bright green carbons for diacylglyceryl (DAG), yellow-green carbons for the *N*-acyl chain, and dark green carbons for the U-domain. Surface representation (left) and a clipped version (right) shows the active site. The light yellow sphere identifies Cys387Ala. The dashed box identifies the magnified region in (B). (**B**) Interactions between Lnt*Eco* Cys387Ala and P2A. Dashed lines identify hydrogen bonds. Other residues form hydrophobic interactions. Residues in P2A are labeled C1, S2, K3, K4, K5, and K6. (**C**) View into the active site of an alignment of P1A (blue) with P2A (purple). (**D**) Shift in arm 3 in going between states P1A and P2A. (**E**) View into the active site of the M2 mimetic state (purple) aligned with P2A. (**F**) View into the active site of an alignment of P2A with P2B (orange). (**G**) Alignment of P2A with P2B showing the shift in arm 7 that accommodates the different peptide sequences in the TA-BLP products. (**H**) Views of the periplasmic opening into and out of the binding pocket in the M1, P1, M2 mimic, and P2 states. The view taken is schematized in the thumbnail (bottom left). Structures are shown in semitransparent surface representation with substrates and products as sticks (outlined). An opening at the top of the binding pocket in M2 and P2 to accommodate the U-domain is indicated by a dashed line. The outlined substrates and products can be seen extending through the opening, with the rest of the molecules in the active site. In M1 and P1, no such opening exists. The outlined substrate and products remain occluded. Arms 1, 3, and 9 are identified by different colors.

There are three acyl chains in the binding pocket in the actual M2 state. One derives from the thioacyl moiety at the catalytic cysteine, while the other two are from DA-BLP. In the M2 mimic structure, the *S*-TITC alkyl chain takes the place of the thioacyl moiety, and the two monooleins recapitulate the two acyl chains in DA-BLP. Comparing the structure of the M2 mimic with that of P1, as represented by P1A, the *S*-acyl and *S*-TITC chains of P1A and M2, respectively, occupy the same space, while the glyceryl, *sn*-2 ester, and acyl moieties of lyso-PE overlap along most of their length with the monoolein molecule, MO1 (Fig. 5F). The second monoolein (MO2) sits in the pocket further from the catalytic center adjacent to MO1 (Fig. 5, C and D). The long axes of MO1 and MO2 run approximately parallel to one another. With three acyl chains in the pocket, the top of the binding cavity is more open than was seen with the M1 and P1 complexes, both of which have two chains. To accommodate the third chain, arm 3 has reorganized with less of it extending over the binding pocket (Fig. 6H). As a result, a 9-Å by 15-Å opening appears in the top of the pocket. This creates a portal big enough to pipe the extended N-terminal tether of a DA-BLP substrate.

There are more than 100 different BLPs in *E. coli* (*40*), making Lnt highly promiscuous with regard to its DA-BLP substrates. The common feature of these acyl acceptors is a diacylglyceryl moiety. We sought to evaluate the extent of Lnt’s promiscuity by testing it with the smallest conceivable DA-BLP substrate, dipalmitoylglyceryl cysteine (DA-Cys). The assay was run in the presence of POPE, and triacylated cysteine (TA-Cys) was identified as a product by TLC and MS (fig. S13). This indicates that a DA-Cys moiety is all that is required for DA-BLP recognition by Lnt and explains its wide-ranging second substrate promiscuity.

### Enzyme–TA-BLPtide product complex: P2 state

The P2 state has Lnt in complex with the final product, TA-BLP. Representatives of P2 were captured in two ways. First, when the Cys387Ser mutant was incubated with FP2, the TA-BLPtide product, FP3, was generated. FP3 remained in the binding pocket, facilitating the structure determination of the Lnt Cys387Ser-FP3 complex at a resolution of 3.0 Å by cryo-EM (P2B; Fig. 2A, figs. S5 and S6, and table S2). Separately, the inactive Cys387Ala mutant form was incubated with Pam_3_CSK_4_ and a cryo-EM structure of the Lnt-Pam_3_CSK_4_ complex was obtained at a resolution of 2.9 Å (P2A; Fig. 2A, figs. S5 and S6, and table S2). The two structures are superimposable with an average RMSD value of 0.66 Å for α carbons (figs. S11 and S12). Because map quality is better for P2A, it will be used to describe the P2 state.

The overall architecture of the protein in the P2A complex closely resembles that observed with representatives of earlier reaction states (Fig. 6A). However, the bound TA-BLPtide sets the P2 state apart. The product sits in the binding pocket where the chain at the catalytic cysteine is transferred to the N-terminal α-amino group of the incoming DA-BLPtide substrate. Accordingly, the acyl amide linkage in TA-BLPtide is positioned within a few angstroms of the catalytic site represented, in this instance, by Cys387Ala (Fig. 6B). The diacylated glyceryl moiety extends away from the catalytic center toward the front of the enzyme. Its acyl chains run parallel to one another and parallel to the *N*-acyl chain, which sits along the bottom of the hydrophobic groove at the base of the pocket (Fig. 6, A and B). The peptide part of the product is fully extended, and the density for the peptide is continuous up to the backbone of the third lysine. The peptide passes through the opening at the top of the binding pocket created by changes in the conformation of arm 3 and arm 7 relative to those adopted in the M1 and P1 states where the much smaller PE or lyso-PE molecules are bound. In size and location, the opening in the pocket is similar to the one observed in the M2 mimic, supporting our denoting this structure as an M2 mimetic (Fig. 6H).

**Fig. 7.**
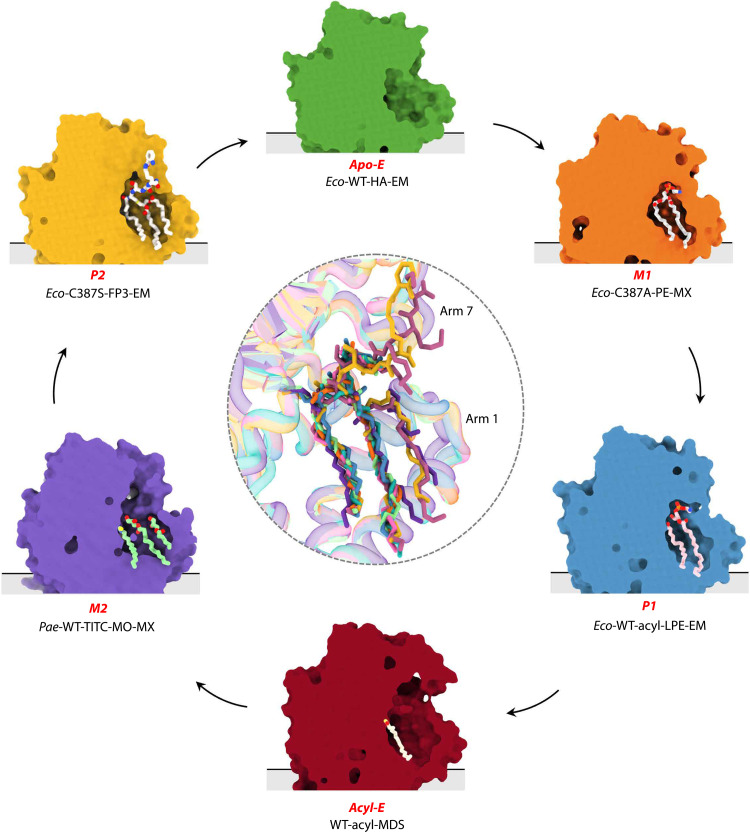
Structural states of Lnt captured throughout the *N*-acyltransferase reaction cycle. The Lnt ping-pong reaction mechanism in structural detail. A clipped view of each of the states revealing the active site region of interest is shown. An alignment of all structures generated in this study is shown at the center with the focus on bound substrates and products. The considerable overlap between substrates and products in this superposition points to a binding pocket in Lnt that can orient very different substrate and product molecules for effective progress through the *N*-acyltransferase reaction cycle. See related movie S2.

A comparison of the P1 and P2 state structures shows that the two protein parts differ mostly in arms 1, 3, and 9 (figs. S11 and S12). The biggest of these is in arm 3 (Fig. 6D). Here, a loop (residues 358 to 363), which in the P1 (and M1) state coordinates with the ammonium of the PE headgroup, rearranges. In doing so, it creates an opening in the binding pocket through which the peptide part of the product extends (Fig. 6H). An overlay of the lipid moieties of the two complexes shows that the thioester-linked acyl chain of P1 and the amide-linked acyl chain of P2 superpose along their length (Fig. 6C). Likewise, the *sn*-2 chain of lyso-PE in P1 superposes on the *sn*-2 chain of the diacylglyceryl in Pam_3_CSK_4_. The *sn*-1 chain of the diacylglyceryl in the product sits atop and is aligned parallel to its neighboring *sn*-2 chain. Similarly, the acyl chains in Pam_3_CSK_4_ superpose on the two monooleins and the TITC modification in the M2 mimic structure (Fig. 6E). The TITC alkyl chain aligns perfectly with the amide-linked acyl chain, while the *sn*-1 and *sn*-2 chains of the diacylglyceryl in both Pam_3_CSK_4_ and FP3 align with MO2 and MO1, respectively. This further supports the hypothesis that the TITC-modified monoolein-bound complex mimics the M2 state. A superposition of all nine structures observed in this study identifies three distinct acyl chain binding sites in Lnt that are occupied to varying degrees throughout the *N*-acylation reaction (Fig. 7).

Note that the P2A and P2B structural representatives are remarkably similar. The main difference is at the end of arm 7, where the loop (residues 234 to 236) changes orientation in response to the disparate sequence and position in the binding pocket of the peptide parts of the products (Fig. 6, F and G). In Pam_3_CSK_4_, the peptide sequence is Cys-Ser-Lys-Lys-Lys-Lys, while in FP3, it is Cys-Ser-Ser-Lys(Abz) (Fig. 3A). The three hydrocarbon chains in the two structures superpose essentially over their full length. Together, these data attest to the similarity of the two representative structures of the P2 state, which further provide a molecular basis for understanding the BLP substrate promiscuity of Lnt.

## DISCUSSION

### Structure snapshots divulge the Lnt *N*-acyltransferase process

Lnt catalyzes the last step in the canonical BLP posttranslational modification pathway. The enzyme converts DA-BLP to TA-BLP using a GPL. In so doing, it prepares BLPs for trafficking to the outer membrane and switches the TLR systems they activate. In this study, we have used the complementary strengths of MX and cryo-EM to elucidate, in molecular detail, how Lnt performs the *N*-acyltransferase reaction by a double-displacement mechanism and how it functions promiscuously with different GPL and BLP substrate types. Nine models have been generated (Fig. 2A), which chart the high-resolution structures and structural changes undergone during the progress of the enzyme, its substrates, products, and intermediates through the *N*-acyltransferase reaction (Fig. 7). We have identified a single active site that has evolved to only bind, individually and sequentially, GPL and BLP substrates that satisfy strict structural and chemical criteria and that can orient in ways defined by the architecture of the binding pocket to position reactive parts next to the catalytic triad for reaction (Fig. 7 and movie S2). The structures explain the role played by many of its essential and nonessential residues and identify arm 3 as a mediator of substrate entry and product egress and, by extension, of catalysis (fig. S10). A molecular understanding of the mechanistic enzymology of the Lnt-catalyzed reaction provided here should facilitate structure-informed design of molecular probes and of antibiotics where off-target effects, on TLRs in particular, are minimized.

## MATERIALS AND METHODS

### Protein expression and purification

The genes for Lnt*Eco* and Lnt*Pae* expression were synthesized and cloned into the pET28a vector using the restriction sites Nde I and Xho I to produce constructs with an N-terminal, thrombin-cleavable His_6_-tag (GenScript, USA). For Lnt*Eco* Cys387Ser and Lnt*Eco* Cys387Ala, the amino acid substitutions were introduced by polymerase chain reaction–based site-directed mutagenesis as per the manufacturer’s instructions (Q5 site-directed mutagenesis kit, New England Biolabs). Information regarding primers used for mutagenesis is in table S3. Expression was carried out in chemically competent C43(DE3) and C41(DE3) cells for Lnt*Eco* and Lnt*Pae*, respectively. Cells were transformed with the desired pET28a-Lnt plasmid and grown in kanamycin-supplemented (50 μg/ml) LB agar plates at 37°C. After a 16-hour incubation, all transformed colonies were suspended in 6 ml of LB. One milliliter of the suspension was used to inoculate 1 liter each of LB or Terrific broth for Lnt*Eco* and Lnt*Pae*, respectively. Cultures were grown at 200 rpm and 37°C to an optical density at 600 nm of 1.6 and then cooled on ice for 20 min. Expression of Lnt was induced by addition of isopropyl-β-d-thiogalactoside to a final concentration of 0.5 mM. Cells were grown for a further 20 hours at 20°C and 200 rpm of shaking postinduction. Cells were harvested by centrifugation at 6000*g* for 10 min at 4°C, resuspended in buffer A [20 mM tris-HCl (pH 8.0), 50 mM NaCl, 0.5 mM EDTA, and 1 mM phenymethylsulfonyl fluoride (PMSF)], and lysed on ice by passing the sample three times through an EmulsiFlex-C5 homogenizer (Avestin, Canada) at 1200 to 1400 bar.

For purification in lauryl maltose neopentyl glycol (LMNG; Generon, UK), membranes were separated from the lysed cell homogenate by centrifugation at 120,000*g* for 45 min at 4°C. The membrane pellet was resuspended in buffer B [20 mM Hepes-NaOH (pH 7.5), 200 mM NaCl, 10% (v/v) glycerol, and 1 mM PMSF) using a Dounce homogenizer at a ratio of 5 ml of buffer per gram of membrane pellet. LMNG was added to a final concentration of 2.5% (w/v), and the membranes were solubilized for 45 min at room temperature (RT; 20°C to 22°C) on a rotating wheel mixer at 12 rpm. The suspension was centrifuged at 60,000*g* for 45 min at 4°C to pellet the unsolubilized material. The supernatant was supplemented with imidazole-HCl from a 2 M stock at pH 7.5 to a final concentration of 20 mM and incubated with 3 ml of Ni-NTA Superflow resin (QIAGEN, Germany) pre-equilibrated in buffer B with 20 mM imidazole and 0.05% (w/v) LMNG. The sample was incubated with the beads on a rotating wheel mixer at 12 rpm for 1 hour at 4°C. The suspension was loaded into an empty chromatography column and the liquid was allowed to drain from the beads under gravity. The beads were washed with 100 ml of buffer C [20 mM Hepes-NaOH (pH 7.5), 800 mM NaCl, 40 mM imidazole, 10% (v/v) glycerol, 0.5 mM PMSF, and 0.05% (w/v) LMNG], and the protein was eluted with 15 ml of buffer D [40 mM sodium citrate (pH 6.0), 200 mM NaCl, 400 mM imidazole, 10% (v/v) glycerol, 0.5 mM PMSF, and 0.1% (w/v) LMNG]. The protein was further purified using a HiLoad 16/60 Superdex 200 column (GE Healthcare, UK) equilibrated in buffer E [20 mM sodium citrate (pH 6.0), 200 mM NaCl, 10% (v/v) glycerol, and 0.05% (w/v) LMNG] at 4°C. The purified protein was concentrated in a 50-kDa molecular weight cut off (MWCO) Amicon Ultra 15 concentrator (Millipore, USA) to ≥13 mg/ml. The purified protein was aliquoted, snap-frozen in liquid nitrogen, and stored at −72°C until required.

For purification in *n*-dodecyl β-d-maltoside (DDM; Sigma-Aldrich), as described by Noland *et al.* (*35*), the membrane pellets were prepared as outlinedabove and resuspended in buffer F [20 mM tris-HCl (pH 8.0), 200 mM NaCl, 10% (v/v) glycerol, and 1 mM PMSF] at 5 ml of buffer per gram of membrane pellet using a Dounce homogenizer. DDM was added to a final concentration of 2% (w/v). The membranes were solubilized for 45 min at RT on a rotating wheel mixer at 12 rpm. The unsolubilized material was removed by centrifugation at 60,000*g* for 45 min at 4°C. The supernatant was supplemented with imidazole-HCl from a 2 M stock at pH 7.5 to a final concentration of 20 mM and mixed with 3 ml of Ni-NTA Superflow resin (QIAGEN, Germany) pre-equilibrated in buffer F with 20 mM imidazole and 0.02% (w/v) DDM. The sample was allowed to bind to the beads at 4°C on a mixer at 12 rpm for 1.5 hours. The suspension was loaded into an empty chromatography column and washed with 100 ml of buffer G [20 mM tris-HCl (pH 8.0), 300 mM NaCl, 10% (v/v) glycerol, 40 mM imidazole, and 0.02% (w/v) DDM]. The protein was eluted with 15 ml of buffer H [20 mM tris-HCl (pH 8.0), 200 mM NaCl, 10% (v/v) glycerol, 400 mM imidazole, and 0.02% (w/v) DDM]. The protein was further purified using a HiLoad 16/60 Superdex 200 column (GE Healthcare, UK) equilibrated with buffer I [20 mM tris-HCl (pH 8.0), 250 mM NaCl, 5% (v/v) glycerol, and 0.02% (w/v) DDM] at 4°C. The purified protein was concentrated in a 50-kDa MWCO Amicon Ultra 15 concentrator (Millipore, USA) to ≥10 mg/ml, aliquoted, snap-frozen in liquid nitrogen, and stored at −72°C until required.

### HA treatment to deacylate Lnt and to deplete it of endogenous GPLs

To generate a sample of apo Lnt*Eco* that is devoid of thioacylation and residual GPL for structural and functional studies, a neutral HA depletion protocol was implemented as described by Buddelmeijer and Young (*39*) (fig. S9B). Lnt*Eco* WT was produced as described above in LMNG, up to and including purification by Ni-NTA. The sample was then buffer-exchanged into buffer J [20 mM Hepes-NaOH (pH 7), 200 mM NaCl, and 0.01% (w/v) LMNG] using a disposable PD10 desalting column (Cytivia) as per the manufacturer’s instructions. The protein was further diluted as required to 0.5 to 1 mg/ml using buffer J. A fresh 2 M stock of HA-HCl was prepared from powder in water and adjusted to pH 7.0 using 6 M NaOH. HA was added to the protein sample to a final concentration of 100 mM. The sample was incubated for 3 hours at 21°C, with mixing by pipetting every 30 min. The sample was applied to a Superdex 200 16/60 gel filtration column (GE Healthcare) equilibrated in buffer E. The peak fractions were concentrated in a 50-kDa MWCO Amicon Ultra 15 concentrator (Millipore, USA) to ≥10 mg/ml, aliquoted, flash-frozen in liquid nitrogen, and stored at −72°C until required.

### Modifying the catalytic cysteine of Lnt using TITC

To modify the enzyme with TITC (Fluorochem Ltd.), a solution of the highly hydrophobic isothiocyanate must be prepared fresh for each use. To this end, 50 ml of a buffer saturated with TITC was made by suspending 7.2 μl of TITC in 45 ml of milliQ water with 0.05% (w/v) DDM or LMNG for Lnt*Eco* WT and Lnt*Pae* WT, respectively. The suspension was sonicated (Bransonic 2800 ultrasonic cleaner, Branson) for 15 min at 21°C and placed on a tube rotator at 12 rpm and 4°C for 15 hours. The suspension was again sonicated for 30 min at 21°C. Hepes-NaOH (pH 8.0) and NaCl were added to final concentrations of 20 and 150 mM, respectively, and the final volume was adjusted to 50 ml with milliQ water. Solubilized TITC aliquots were centrifuged immediately before use at 20,000*g* for 5 min at 21°C to remove the insoluble material. The actual concentration of TITC in the aqueous solution used for modification was estimated at between 0.1 and 0.2 mM.

For treatment with TITC, purified and concentrated Lnt*Pae* WT in buffer E was diluted into the freshly prepared TITC-LMNG solution to a final concentration of 10 μM protein. For Lnt*Eco*, WT enzyme in buffer I was diluted into freshly prepared TITC-DDM solution to a final concentration of 10 μM. The samples were incubated for 2 hours at RT with mixing by pipetting every 30 min. After incubation, the samples were transferred to a 50-kDa MWCO Amicon Ultra 15 concentrator (Millipore, USA) and concentrated at 4°C according to the manufacturer’s instructions. The resulting 0.25-ml concentrate was diluted again into a fresh TITC-LMNG (Lnt*Pae*) or TITC-DDM (Lnt*Eco*) solution to a final concentration of 10 μM Lnt. These were incubated for another 2 hours as described previously. This process was repeated until the protein was incubated with TITC for a total of 6 hours at RT. The sample was concentrated to 0.25 ml and diluted to 10 μM Lnt with fresh TITC-LMNG or TITC-DDM and then incubated for 18 hours at 4°C on a tube rotator at 12 rpm. Samples were buffer-exchanged into buffer E (Lnt*Pae*) or buffer I (Lnt*Eco*) using a PD10 desalting column (Cytivia) as per the manufacturer’s instructions. The protein sample was concentrated to ≥10 mg/ml, aliquoted, flash-frozen in liquid nitrogen, and stored at −72°C until required.

### Lnt activity assays

#### Step 1

The assay was carried out as described by Wiktor *et al.* (*37*). Briefly, 18:1-12:0 NBD-PE [7-nitro-2-1,3-benzoxadiazol-4-yl)amino (NBD)] (Avanti Polar Lipids Inc.) and the commercial DA-BLPtide, FSL-1 (EMC Microcollections, Germany), were incubated with Lnt. The enzyme transfers the oleic acid (18:1) at the *sn*-1 position of the NBD-PE substrate to the free sulfhydryl at the catalytic cysteine, forming NBD-lyso-PE and the thioacylated Lnt. The NBD fluorophore is present in both the lipid substrate and lysolipid product. These are separated by TLC and individually quantified by fluorescence imaging. Assays were carried out in 50 μl of buffer K [50 mM tris-HCl (pH 7.5), 150 mM NaCl, and 0.01% (w/v) LMNG] containing 500 μM NBD-PE and 150 μM FSL-1. The reaction was initiated by adding 10 μM Lnt*Eco* and was carried out at 37°C with shaking at 180 rpm. After 60 min, the reaction was stopped by flash-freezing the samples in liquid nitrogen. To extract NBD-PE and NBD-lyso-PE, 50 μl of 70% (v/v) ethanol was added to the frozen reaction mix followed by vortexing for 10 to 20 s at RT until a homogenous solution was obtained. Thirty microliters of chloroform was added and the sample was vortex-mixed for a further 1 min at RT. Phase separation was facilitated by centrifugation for 2 min at 13,000*g* at RT. The lower chloroform phase containing NBD-PE and NBD-lyso-PE was transferred to a fresh 1.5-ml Eppendorf tube. The tube was left open in a fume hood for 15 min at RT to allow most of the chloroform to evaporate passively, concentrating the lipid. The tube was centrifuged for 2 min at 13,000*g* and at RT, and all of the collected organic phase were spotted on a Silica gel 60 F254 TLC plate (Sigma-Aldrich), pre-run in chloroform. The plate was placed in a desiccator at 21°C under a high vacuum (50 mbar) for 10 min to remove residual dimethyl sulfoxide (DMSO) carried over from the NBD-PE stock solution. Chromatography was carried out with a mobile phase consisting of chloroform:acetone:acetic acid:methanol:water [10:4:2:2:1 (v/v)]. The plate was dried in a stream of nitrogen and imaged using a Biorad Chemidoc MP imager (fluorescein filter). Fluorescent intensity associated with NBD-lyso-PE, and NBD-PE was quantified by image analysis using ImageJ. The results were plotted using PRISM 6.0 (GraphPad).

#### Step 2

The reaction catalyzed by Lnt in step 2 was monitored by tracking the conversion of the in-house synthesized fluorescently labeled lipopeptide substrate, FP2 to FP3, in the presence of GPL (POPE in most cases) as lipid substrate and Lnt. FP2 and FP3 were separated by TLC and quantified individually by fluorescence intensity measurement. The assay was performed in 50 μl of reaction mix containing 500 μM GPL (Avanti Polar Lipids Inc.) and 150 μM FP2 in buffer K [50 mM tris-HCl (pH 7.5), 150 mM NaCl, and 0.01% (w/w) LMNG]. The reaction was initiated by adding 10 μM enzyme, to make a final volume of 50 μL, and was carried out at 37°C with shaking at 180 rpm. For GPL substrate specificity assays, the reaction was allowed to proceed for 90 min before flash-freezing samples in liquid nitrogen at the times indicated (fig. S2). FP2 and FP3 were extracted as described above for NBD-PE and NBD-lyso-PE. The tubes were left open in a fume hood for 10 min at RT to allow most of the chloroform to evaporate passively, concentrating the BLPtides. The tubes were centrifuged for 2 min at 13,000*g* and at RT, and all of the collected organic phase were spotted on a Silica gel 60 F254 TLC plate (Sigma-Aldrich), pre-run in chloroform. The TLC was developed in chloroform:methanol:ammonia_aq_ [9:2:0.01 (v/v)], and the substrate and product were directly visualized on the TLC plate under ultraviolet light at 254 nm. A digital image of the chromatogram was recorded using a smartphone, and substrate and product spot intensities were quantified by image analysis using ImageJ. The results were plotted using PRISM 6.0 (GraphPad).

### Mass spectrometry

Purified Lnt (in DDM, WT, and mutants) were buffer-exchanged into buffer L [200 mM ammonium acetate (pH 7.0) supplemented with 0.5% (w/v) tetraethylene glycol monooctyl ether (C8E4) or 1% (w/v) *n*-octyl-β-d-glucopyranoside]. For incubation with FP2, samples containing FP2 (0.2 mg/ml) and 10 μM Lnt in buffer L were incubated at 22°C for 1 hour before end point measurements. Spectra were acquired on a Q-Exactive hybrid quadrupole–Orbitrap mass spectrometer (Thermo Fisher Scientific, Bremen, Germany). Typically, 2- to 3-μl sample aliquots were loaded into a gold-coated borosilicate capillary (Harvard Apparatus; prepared in-house), and the capillary was mounted on the nano–electrospray ionization (ESI) source. The instrument settings were 1.2-kV capillary voltage, S-lens radio frequency (RF) 200%, quadrupole selection from 1000 to 10,000 mass/charge ratio range, argon ultra high vacuum (UHV) pressure of 3.3 × 10^−13^ bar, and capillary temperature 150°C. Unless otherwise stated, the resolution of the instrument was set to 17,500 at a transient time of 64 ms. Voltages of the ion transfer optics—injection flatapole, interflatapole lens, bent flatapole, and transfer multipole—were set to 5, 3, 2, and 30 V, respectively. The noise level was set at 3.

Co-purified lipids were extracted by mixing 50 μl of 20 μM protein sample in buffer L with 200 μl of chloroform/methanol [2:1 (v/v) mixtures]. The bottom organic layer was separated by centrifugation at 20,000*g* for 30 min at 4°C, removed, and then dried under vacuum at 45°C for 2 hours. The resulting lipid film was resuspended in a buffer of 60% (v/v) acetonitrile containing 10 mM ammonium formate and 0.1% (v/v) formic acid. The samples were analyzed in the negative ESI polarity on an Eclipse Tribrid mass spectrometer (Thermo Fisher Scientific) using a source activation of 175 V for MS^1^, supplemented with an additional 28 to 32 V via resonant collision-induced dissociation for fragmentation MS^2^. For the Lnt-mediated acylation of DA-Cys, samples were prepared and extracted as outlined above for the step 2 activity assay, replacing FP2 with DA-Cys. Five microliters of the chloroform extract of the reaction mixture was dried under vacuum, and the substrate film was resuspended in 5 μl of DMSO. This was further 10-fold diluted in DMSO before measurement in the positive ESI polarity (peptide mode) on the Eclipse Tribrid mass spectrometer. Data were visualized and exported for processing using the Qual browser of Xcalibur 4.1.31.9 (Thermo Fisher Scientific), and spectral deconvolution was performed using UniDec software (*41*). All measurements were performed at least three times and yielded similar results.

### Cryo-EM, model building, and refinement

A total of 200-μl aliquots of the desired Lnt (HA-treated Lnt*Eco* WT for the apo structure, Cys387Ala or Cys387Ser for M1 complexes, and Lnt*Eco* WT for the P1 complex) at ≥10 mg/ml in buffer E were defrosted on ice. For the P2 structures with Pam3CSK4 and FP3, 60 μl of Pam3CSK4 or FP2 (10 mg/ml) in pure DMSO was added to 140 μl of Lnt*Eco* Cys387Ala or Lnt*Eco* Cys387Ser (16 mg/ml), respectively. The mixtures were incubated for 1 hour at 21°C, and the entire 200-μl sample was loaded onto a Superdex 200 10/300 Increase (GE Healthcare) column pre-equilibrated with buffer M [20 mM sodium citrate (pH 6), 150 mM NaCl, and 0.001% (w/v) LMNG]. The peak fractions were pooled and concentrated in a 100-kDa cutoff MWCO Amicon Ultra 15 concentrator (Millipore, USA) to 14 to 17 mg/ml (fig. S5, A and B). A total of 3.5 μl of the sample was applied to glow-discharged UltrAuFoil grids (UltrAuFoil R1.2/1.3, 300 mesh, 1.5 min, 15 mA; PELCO easiGlow, TED PELLA). The grids were blotted (4.0- to 4.5-s blot, 10-s wait time, and −1 blot force at 22°C and 100% relative humidity) using a Vitrobot Mark IV (Thermo Fisher Scientific) and plunge-vitrified in liquid ethane. Movies were recorded on a Titan Krios electron microscope (Thermo Fisher Scientific) equipped with a K3 direct electron detector (Gatan), operating at 300 kV at a calibrated fold-magnification of 105,000 corresponding to a magnified pixel size of 0.826 Å (see table S2 for details regarding the number of movies collected per dataset). The BioQuantum energy filter (Gatan) was operated with an energy slit width of 20 eV. Micrographs were recorded at an exposure rate of ∼22 electrons/(Å^2^·s), and defocus values ranging from −0.8 to −2.2 μm. The total exposure time was 1.49 s, and intermediate frames were recorded in 33-ms intervals, resulting in an accumulated dose of ∼33.0 electrons/Å^2^ and a total of 45 frames per micrograph. Automatic data acquisition was done using E Pluribus Unum (EPU, Thermo Fisher Scientific).

Patch motion correction, contour transfer function (CTF) estimation, particle picking, 2D classification, ab initio model reconstruction, and heterogeneous refinement were performed in cryoSPARC v3.1 (*42*). Homogeneous groups of particles with well-resolved features were subsequently subjected to nonuniform refinement. Particle coordinates with their assigned angles were imported into Relion 3.1 (*43*) and were subjected to alignment-free 3D classification to enrich for more refined homogeneous particle populations. Particles that corresponded to classes with well-defined density were transferred back to cryoSPARC 3.1 and were subjected to a nonuniform refinement followed by a local refinement focused on the protein excluding the micelle and a CTF refinement. Details regarding number of particles used for each dataset are provided in table S2. A flowchart describing data processing steps is in fig. S5. These procedures yielded final maps with indicated global resolutions of 3.0, 3.0, 3.1, 2.7, 2.9, and 3.0 Å for apo, M1B, M1C, P1A, P2A, and P2B structures, respectively. The indicated resolution values are based on the gold-standard Fourier shell correlation using the 0.143 criterion (fig. S5F). Angular distribution for particle projections was plotted using cryoSPARC v3.2 (fig. S6A) (*42*). Local resolution was calculated in cryoSPARC v3.2 (*42*) and plotted using ChimeraX (fig. S6G) (*44*).The initial coordinates of Lnt*Eco* were generated using the Lnt*Eco* Cys387Ala crystal structure, Protein Data Bank (PDB) 5N6L (*37*). Ligand coordinates and geometry restraints were generated using GradeWebServer (*45*). Models were initially docked into the EM density map using UCSF Chimera (*46*), followed by iterative manual building in Coot (*47*). The final models were subjected to global refinement and minimization in real space using phenix.real_space_refine implemented in Phenix (*48*). MolProbity (*49*) was used to evaluate model geometry. The final refinement parameters are provided in table S2. ChimeraX (*44*) was used for figure preparation. Structures have been deposited into the PDB and Electron Microscopy Data Bank (EMDB) databases with accession codes 8B0K/EMD-15786 (apo), 8B0M/EMD-15788 (M1B), 8B0L/EMD-15787 (M1C), 8B0N/EMD-15789 (P1A), 8B0P/EMD-15791 (P2A), and 8B0O/EMD-15790 (P2B).

### Crystallization

The Lnt*Eco*-PE complex (M1A) was prepared following the Lnt crystallization protocol of Noland *et al.* (*35*) with some modifications. Briefly, 12 μl of a solution (25 mg/ml) of *E. coli* polar lipids (Avanti) in chloroform was placed in a 0.5-ml Eppendorf tube. The chloroform was evaporated by centrifuging the open tube at RT, 4000*g* for 16 hours at 1.3 mbar to coat the wall of the tube with a thin lipid film. A total of 25 μl of Lnt*Eco* Cys387Ala (10 mg/ml) in buffer I was added to the tube and incubated for 16 hours at 4°C with mixing at 12 rpm on a tube rotator. The insoluble material was removed by centrifugation for 30 min at 20,000*g* and at 4°C. The supernatant was transferred to a new Eppendorf tube and was used directly for vapor diffusion crystallization. Crystallization drops were set up in 24-well plates by combining 1 μl of precipitant [50 mM sodium acetate (pH 5.0), 50 mM magnesium acetate, and 28 to 36% (v/v) polyethylene glycol, molecular weight 200 (PEG200)] and 1 μl of the Lnt*Eco* Cys387Ala/lipid mix on silanized glass coverslips. Drops were equilibrated against 1 ml of precipitant solution at 18°C. After 2 weeks, crystals had grown to about 30 μm by 30 μm by 500 μm (movie S1). Crystals were loop-harvested and snap-cooled in liquid nitrogen directly and without added cryo-protectant.

In an effort to crystallize a complex between TITC-treated Lnt*Eco* and FP2, 12 μl of *E. coli* polar lipids (Avanti) at 25 mg/ml and 50 μl of FP2 at 10 mg/ml (both in chloroform) were combined in a 1.5-ml Eppendorf tube. The chloroform was evaporated as described above for the M1A complex. A total of 25 μl of TITC-treated Lnt*Eco* at 10 mg/ml in buffer I was transferred into the Eppendorf tubes containing *E. coli* polar lipids and FP2. The sample was incubated for 16 hours at 4°C with gentle agitation. The insoluble material was separated by centrifugation for 30 min at 20,000*g* and 4°C. The supernatant was transferred to a fresh Eppendorf tube and was used directly for vapor diffusion crystallization as outlined above for the M1A complex. As described in the section “Acyl-enzyme–lipid product complex: P1 state,” the protein crystallized in the same space group as the Lnt-PE complex. However, instead of FP2, a lyso-PE molecule, which presumably came from the *E. coli* polar lipids, was found in the active site of the enzyme. This structure was chosen to represent Lnt in the P1 state (P1B).

For in meso crystallization trials, the TITC-treated Lnt*Pae* protein was reconstituted into the bilayer of the cubic mesophase following a standard protocol (*50*, *51*). The protein solution was homogenized with monoolein [9.9 monoacylglycerol (MAG)] in a coupled syringe mixing device using two volumes of protein solution and three volumes of lipid (*52*). Crystallization trials were set up by transferring 50 nl of the protein-laden mesophase onto a silanized 96-well glass sandwich plate followed by 800 nl of precipitant solution using an in meso robot (*53*). The glass plates were sealed and then stored in an imager (RockImager 1500, Formulatrix, USA) at 20°C for crystal growth. Crystals of TITC-treated Lnt*Pae* were obtained with 30% (v/v) PEG-500 dimethyl ether, 0.1 M sodium citrate (pH 5.0), and 0.1 M sodium acetate. Lnt*Pae*-TITC crystals were grown and data were collected using the in meso in situ serial x-ray crystallography method, as described (*54*).

### Macromolecular x-ray crystallography

#### Diffraction data collection and processing

X-ray diffraction experiments were carried out at 100 K for both M1A *Eco*-Cys387Ala-PE and P1B *Eco*-WT-TITC-LPE on protein crystallography beamline I24 at the Diamond Light Source, UK. Measurements were made in steps of 0.2° at a speed of 2°/s with the PILATUS 6M-F detector operating in the continuous/shutterless data collection mode at a sample-to-detector distance of 35 to 40 cm. Diffraction data were collected at a wavelength of 0.96861 Å and at 1.731 × 10^12^ to 1.883 × 10^12^ photons/s.

Serial data collection for M2 *Pae*-WT-TITC-MO was carried out at 100 K on the protein crystallography beamline X06SA-PXI at the Swiss Light Source, Villigen, Switzerland. Measurements were made in steps of 0.1° at a speed of 1°/s using the data acquisition software DA+ (*55*), an automated serial data collection protocol (*54*, *56*), and with the EIGER 16M detector operated in a continuous/shutterless data collection mode. Data were collected at a wavelength of 1.0 Å, a flux of 1.385 × 10^12^ photons/s, and a sample-to-detector distance of 40 cm.

Data for both M1A *Eco*-Cys387Ala-PE and M2 *Pae*-WT-TITC-MO were processed with XDS and scaled and merged with XSCALE (*57*). Data for P1B *Eco*-WT-TITC-LPE were processed with autoPROC 1.0.5/STARANISO (*58*, *59*). The dataset for M1A *Eco*-Cys387Ala-PE, to a resolution of 2.40 Å, was obtained with a single crystal over an angular range of 100°. For P1B *Eco*-WT-TITC-LPE, a dataset to a resolution of 2.66 Å was obtained from one crystal collected over 180°. The IMISX serial dataset for M2 *Pae*-WT-TITC-MO was merged from 41 datasets collected over an angular range of 10° to 30° to a resolution of 2.6 Å. Data collection parameters are summarized in table S1.

#### Structure determination and refinement

The structures were solved by molecular replacement using the program Phaser (*60*) with PDB 5N6L (*37*) as search model. BUSTER (*61*) and PHENIX.refine (*48*) were used during the refinement of all structures. Coot (*47*) was used for manual structure building. Refinement statistics are reported in table S1. Figures were generated using ChimeraX (*44*). Structures and associated structure-factor amplitudes have been deposited in the PDB with accession codes 8AQ3 (M1A), 8AQ4 (P1B), and 8AQ2 (M2).

### Quantifying the phospholipid content of purified Lnt

To estimate the number of GPL molecules co-purified with Lnt*Eco* WT, the total phosphorous content of the protein sample was measured. The assay involved wet ashing to convert organic phosphorous, primarily from bound GPLs to inorganic phosphate, which was quantified colorimetrically. The procedure involved washing 13-mm × 100-mm Fisher brand glass tubes with 1 ml of 1% (v/v) nitric acid three times, extensive rinsing with milliQ water, and drying in an oven at 120°C for 20 min. Samples of purified protein were added to the tubes in duplicate and dried at 120°C for 10 min. A standard curve using KH_2_PO_4_ at 0, 0.2, 0.4, 0.6, 0.8, and 1 μg of KH_2_PO_4_ in duplicate was prepared in parallel (fig. S9A). To all tubes, 0.13 ml of 70% (w/v) perchloric acid (Sigma-Aldrich) was added, and the tubes were wet ashed in a heat block at 180°C for 50 min. Upon cooling to 20°C, 0.66 ml of milliQ water was added to each tube, followed by 0.1 ml of freshly prepared 2.5% (w/v) ammonium molybdate, and 0.1 ml of 10% (w/v) ascorbic acid. The tubes were heated to 110°C for 7 min and then cooled to RT. A total of 330 μl of each sample was transferred to a 96-well plate for absorbance measurement at 810 nm in a Spectramax M2e plate reader. Phospholipid content was estimated on the basis of the assumption that each GPL molecule has one phosphorous atom and has a molecular weight of 600 kDa (*62*).

## Supplementary Materials

### This PDF file includes:

Supplementary Text Figs. S1 to S17 Legends for tables S1 and S2 Table S3 Legends for movies S1 and S2 Legend for database S1 References

### Other Supplementary Material for this manuscript includes the following:

Movies S1 and S2

Database S1
