# Supplementary material for: Structure snapshots reveal the mechanism of a bacterial membrane lipoprotein N-acyltransferase
Source: Sci Adv. 2023 Jun 30;9(26):eadf5799. doi: 10.1126/sciadv.adf5799 (PMC10313180; doi:10.1126/sciadv.adf5799)
Supplement: Supplementary file 1 — Supplementary Text Figs. S1 to S17 Legends for tables S1 and S2 Table S3 Legends for movies S1 and S2 Legend for database S1 References [file sciadv.adf5799_sm.pdf]

Science Advances
AAAS

Supplementary Materials for

Structure snapshots reveal the mechanism of a bacterial membrane lipoprotein *N*-acyltransferase

Luke Smithers *et al.*

Corresponding author: Moran Shalev-Benami, moransb@weizmann.ac.il; Martin Caffrey, martin.caffrey@tcd.ie

Sci. Adv. **9**, eadf5799 (2023)
DOI: 10.1126/sciadv.adf5799

The PDF file includes:

Supplementary Text
Figs. S1 to S17
Legends for tables S1 and S2
Table S3
Legends for movies S1 and S2
Legend for database S1
References

Other Supplementary Material for this manuscript includes the following:

Tables S1 and S2
Movies S1 and S2
Database S1

Supplementary Text

Chemical synthesis and compound characterisation

Abbreviations

Abz	2-aminobenzoyl
Ar	aromatic
calcd.	calculated
d	doublet
DAG	diacylglyceryl
dd	doublet of doublets
DIPEA	*N*,*N*-diisopropylethylamine
DMAP	4-(dimethylamino)pyridine
DMF	*N*,*N*-dimethylformamide
EDC·HCl	*N*-(3-dimethylaminopropyl)-*N′*-ethylcarbodiimide hydrochloride
Equiv.	equivalents
ESI	electrospray ionization
EtOAc	ethyl acetate
Fmoc	fluorenylmethyloxycarbonyl
HOBt	1-hydroxybenzotriazole monohydrate
HRMS	high resolution mass spectrometry
IR	infrared
m	multiplet
MALDI	matrix assisted laser desorption ionization
MeOH	methanol
NMM	*N*-methylmorpholine
NMR	nuclear magnetic resonance

Mtt	4-methyltrityl
Pal	palmitic
Pam	pamitoyl
ppm	parts per million
PyBOP	benzotriazol-1-yl-oxytripyrrolidinophosphonium hexafluorophosphate
qC	quaternary carbon
Q-ToF	quadrupole time-of-flight
s	singlet
SPPS	sold phase peptide synthesis
t	triplet
TES	triethylsilane
TFA	trifluoroacetic acid
TLC	thin layer chromatography
R_f	retention factor

Instrumental and general considerations

Proton nuclear magnetic resonance (^{1}H NMR) and carbon nuclear magnetic resonance (^{13}C NMR) spectra were recorded on a 400 MHz Bruker Advance spectrometer, ^{1}H (400.13 MHz) and ^{13}C (100.6 MHz) or a 600 MHz Bruker Avance II spectrometer, ^{1}H (600.13 MHz) and ^{13}C (150.6 MHz). Resonances δ, are in parts per million (ppm) calibrated using residual undeuterated solvent (^{1}H NMR) or the deuterated solvent (^{13}C NMR) as internal reference standards. Infrared (IR) spectra were recorded on a Perkin Elmer spectrometer. Mass spectrometry analysis was performed with a Waters Premier quadrupole time-of-flight (Q-ToF) mass spectrometer equipped with Z–spray electrospray ionization (ESI) and matrix assisted laser desorption ionization (MALDI) sources. Silica gel Florisil (200 mesh; Aldrich)

was used for column chromatography. Thin-layer chromatography (TLC) was performed using Merck 60 F254 silica gel plates (pre-coated, 0.2 mm thick) and visualized by UV light (λ_{max} = 254 nm) and ammonium molybdate staining (ammonium molybdate (0.26 M) in aq. H_2SO_4 (1 M)). Unless otherwise stated, protected amino acids for peptide synthesis and all other reagents were purchased from an industrial supplier. ^{1}H NMR and ^{13}C NMR spectra of novel compounds are included as Supplementary Figures. In the following section, the target compounds and the intermediates used in their syntheses are identified in shorthand by emboldened arabic numerals. Peptide synthesis was performed using manual Fmoc/*t*Bu solid phase peptide synthesis (SPPS) in polypropylene syringe reaction vessels (10 mL; Torviq, MI, USA). All SPPS reactions were carried out at room temperature (19.5 – 20.5°C) under continuous agitation.

Synthesis of Diacylated Cysteine Derrivative (DA-Cys)

DA-Cys was synthesized using an adapted literature procedure as shown in **Fig. S14**.

Methyl *N*-(*tert*-butoxycarbonyl)-*S*-(2,3-dihydroxypropyl)-L-cysteinate (**2**)

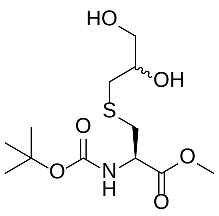

To a solution of Fmoc-Cys-OMe (**1**) (1.46 g, 6.23 mmol) in anhydrous *N*,*N*-dimethylformamide (DMF) (10 mL) under argon at room temperature was added Cs_2CO_3 (2.03 g, 6.23 mmol). The reaction was stirred at room temperature for 1 h. A solution of 3-bromo-1,2-propandiol (1.06 g, 6.85 mmol) in anhydrous DMF (5 mL) under argon and was then added to the reaction which was stirred at room temperature for 16 h. H_2O (100 mL)

was added and the product was extracted with CH_2Cl_2 (3 x 100 mL). The combined organic layers were washed with brine (5 x 100 mL), dried over $MgSO_4$ and filtered. The solvent was removed *in vacuo* and the crude product was purified by silica column chromatography (EtOAc:*n*-hexane, 8:2) to yield a yellow oil (1.22 g, 63%).

TLC (EtOAc:*n*-hexane 8:2) R_f = 0.25; ^{1}H NMR (400 MHz, $CDCl_3$) two diastereomers (1:1) δ 5.46 – 5.39 (m, 1H, NH), 4.62 – 4.51 (m, 1H, αCH), 3.85 – 3.78 (m, 1H, S-glyceryl CH), 3.77 (s, 3H, OCH_3), 3.74 – 3.68 (m, 1H, S-glyceryl $OC\underline{H}_aH_b$), 3.59 – 3.52 (m, 1H, S-glyceryl $OCH_a\underline{H}_b$), 3.05 – 2.98 (m, 1H, $\beta C\underline{H}_aH_b$), 2.95 – 2.86 (m, 1H, $\beta CH_a\underline{H}_b$), 2.84 – 2.72 (m, 1H, S-glyceryl $C\underline{H}_aH_b$) 2.68 – 2.59 (m, 1H, S-glyceryl $CH_a\underline{H}_b$), 1.45 (s, 9H, Boc CH_3); ^{13}C NMR (101 MHz, $CDCl_3$); two diastereomers (1:1) δ 171.7 (αCH-$\underline{C}$O, diastereomer A), 171.6 (αCH-$\underline{C}$O, diastereomer B), 155.6 (NH-CO), 80.6 (Boc qC), 70.9 (S-glyceryl CH, diastereomer A), 70.7 (S-glyceryl CH, diastereomer B) 65.3 (S-glyceryl OCH_2, diastereomer A), 65.2 (S-glyceryl OCH_2, diastereomer B), 53.6 (αC), 52.8 (OCH_3), 36.4 (S-glyceryl CH_2), 35.7 (βC, diastereomer A), 35.5 (βC, diastereomer B), 28.4 (Boc CH_3); HRMS (ESI$^+$) calcd. $C_{12}H_{23}NNaO_6S$ = 332.1138 $(M + Na)^+$. Found = 332.1133; ν_{max} (thin film)/cm^{-1} 3373 (OH), 2977 (CH), 1741 (ester C=O), 1689 (Boc C=O).

3-(((*R*)-2-((*tert*-butoxycarbonyl)amino)-3-methoxy-3-oxopropyl)thio)propane-1,2-diyl dipalmitate (**3**)

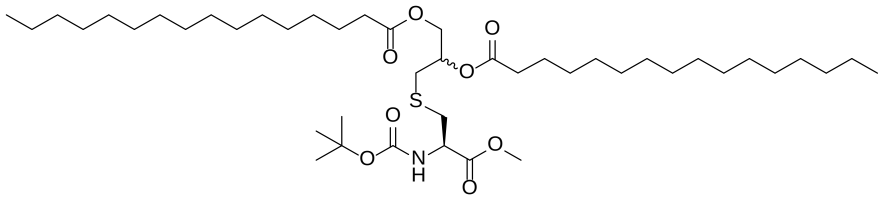

To a solution of palmitic acid (518 mg, 2.02 mmol) in anhydrous THF:CH_2Cl_2 (2:3, 20 mL) at 0°C under argon was added EDC·HCl (465 mg, 2.43 mmol) and DMAP (10 mg, 0.081 mmol). The reaction was stirred at 0°C for 1 h. Diol **2** (250 mg, 0.808 mmol) dissolved in anhydrous THF (5 mL) was added to the reaction which was stirred at room temperature under argon for 3 days. The solvents were removed *in vacuo* and the crude product was purified by silica column chromatography (EtOAc:*n*-hexane, 3:7) to yield the diacylated compound **3** as a white solid (444 mg, 70%). The isolated compound was in good agreement with the literature (*63*).

TLC (EtOAc:*n*-hexane 35:65) R_f = 0.64; ^{1}H NMR (400 MHz, $CDCl_3$) two diastereomers (1:1) δ 5.36 – 5.27 (m, 1H, NH), 5.15 – 5.08 (m, 1H, Cys αCH), 4.56 – 4.49 (m, 1H, S-glyceryl CH), 4.33 – 4.28 (m, 1H, S-glyceryl $OC\underline{H}_aH_b$), 4.16 – 4.10 (m, 1H, S-glyceryl $OCH_a\underline{H}_b$), 3.74 (s, 3H, OCH_3), 3.11 – 2.89 (m, 2H, Cys βCH_2), 2.77 – 2.66 (m, 2H, S-glyceryl CH_2), 2.38 – 2.20 (m, 4H, Pal αCH_2), 1.63 – 1.54 (m, 4H, Pal βCH_2), 1.43 (s, 9H, Boc CH_3), 1.33 – 1.18 (m, 48H, Pal CH_2), 0.86 (t, *J* = 6.8 Hz, 6H, Pal CH_3); *m/z* HRMS (ESI^+) calcd. $C_{44}H_{83}NO_8SNa$ = 808.5741 $(M + Na)^+$. Found = 808.5732

<u>3-(((*R*)-3-methoxy-3-oxo-2-((2,2,2-trifluoroacetyl)-λ^4-azaneyl)propyl)thio)propane-1,2-diyl dipalmitate (**4**)</u>

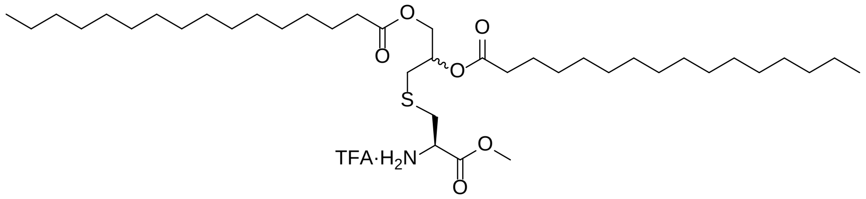

Compound **3** (62 mg, 0.079 mmol) was dissolved in CH_2Cl_2 (3 mL) and trifluoroacetic acid (TFA) (1 mL) was added. The reaction was stirred at room temperature for 16 h and the

solvents removed *in vacuo* to yield a pale yellow solid (57 mg, 90%). The isolated compound was in good agreement with the literature (*62*).

TLC (EtOAc:*n*-hexane 1:1) R_f = 0.26; ^{1}H NMR (400 MHz, $CDCl_3$) two diastereomers δ 5.22 – 5.13 (m, 1H, S-glyceryl CH), 4.43 – 4.29 (m, 2H, Cys αCH, S-glyceryl OC$\underline{H}_aH_b$), 4.21 – 4.10 (m, 1H, S-glyceryl OC$H_a\underline{H}_b$), 3.88 (s, 3H, OCH_3), 3.39 – 3.14 (m, 2H, βCH_2), 2.85 – 2.72 (m, 2H, S-glyceryl CH_2), 2.41 – 2.30 (m, 4H, Pal αCH_2), 1.69 – 1.57 (m, 4H, Pal βCH_2), 1.38 – 1.19 (m, 48H, Pal CH_2), 0.90 (t, *J* = 6.9 Hz, 6H, Pal CH_3); ^{19}F NMR (377 MHz, $CDCl_3$) two diastereomers δ -75.91; *m/z* HRMS (ESI$^+$) calcd. $C_{39}H_{76}NO_6S$ = 686.5388 $(M + H)^+$. Found = 686.5380.

Peptide Synthesis

FP2 was synthesized as previously described (*64*) and as outlined in **Fig. S15.** The protected amino acid 2-((*tert*-butoxycarbonyl)amino)benzoic acid (Boc-Abz-OH) and the modified cysteine building block *N*-(((9*H*-fluoren-9-yl)methoxy)carbonyl)-*S*-((*R*)-2,3-bis(palmitoyloxy)propyl)-L-cysteine (Fmoc-Cys((*R*)-2,3-bis(palmitoyloxy)propyl)-OH) required for peptide synthesis were synthesized as previously reported (*22*).

FP2 - (7*S*,10*S*,13*S*,16*R*,20*R*)-16-Amino-1-(2-aminophenyl)-7-carbamoyl-10,13-bis(hydroxymethyl)-1,9,12,15-tetraoxo-18-thia-2,8,11,14-tetraazahenicosane-20,21-diyl dipalmitate (**10**)

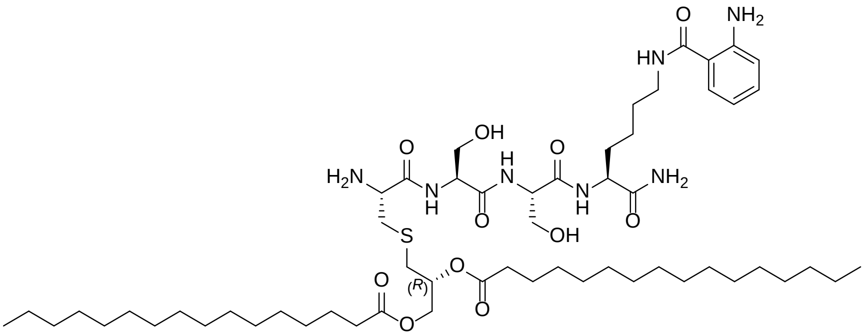

To a polypropylene syringe reaction vessel was added rink amide (aminomethyl)polystyrene resin (209 mg, 0.150 mmol; initial loading 0.70 mmol/g) and DMF (5 mL). The syringe was agitated for 20 min and then drained. A solution of 20 %(v/v) piperidine in DMF (5 mL) was added for 2 x 10 min. The Fmoc deprotected resin was drained and washed with DMF (3 x 5 mL), CH_2Cl_2 (3 x 5 mL) and DMF (3 x 5 mL). A solution of Fmoc-Lys(N^{ε}-4-methyltrityl)-OH (4 equiv, 365 mg, 0.580 mmol), PyBOP (4 equiv, 304 mg, 0.580 mmol) and NMM (8 equiv, 0.129 mL, 1.17 mmol) in DMF (3 mL) was added to the resin and agitated for 45 min. The reaction vessel was drained and the resin was washed with DMF (3 x 5 mL), CH_2Cl_2 (3 x 5 mL), DMF (3 x 5 mL) and CH_2Cl_2 (3 x 5 mL). A solution of 5% (v/v) TFA, 5% (v/v) triethylsilane (TES), 90 %(v/v) CH_2Cl_2 (5 mL) was added to the resin for 5 x 1 min to remove 4-methyltrityl. The resin was drained and washed with CH_2Cl_2 (3 x 5 mL), DMF (3 x 5 mL), CH_2Cl_2 (3 x 5 mL) and DMF (3 x 5 mL). A solution of Boc-Abz-OH (3 equiv, 104 mg, 0.438 mmol), PyBOP (3 equiv, 228 mg, 0.438 mmol) and NMM (6 equiv, 0.096 mL, 0.876 mmol) in DMF (2 mL) was added to the syringe which was agitated for 45 min then drained and washed with DMF (3 x 5 mL), CH_2Cl_2 (3 x 5 mL) then DMF (3 x 5 mL). Two sequential couplings of Fmoc-Ser(*t*Bu)-OH were then performed on the resin using coupling cycles consisting of (i) Fmoc deprotection using 20% (v/v) piperidine in DMF (2 x 10 min; 5 mL), (ii) resin washing with DMF (3 x 5 mL), CH_2Cl_2 (3 x 5 mL) then DMF (3 x 5 mL), (iii) peptide coupling by the addition of PyBOP (3 equiv, 228 mg, 0.438 mmol), NMM (6 equiv, 0.096 mL, 0.876 mmol) and Fmoc-Ser(*t*Bu)-OH (3 equiv, 168 mg, 0.438 mmol) in DMF (2 mL) to the peptide resin for 45 min, (iv) resin washing with DMF (3 x 5 mL), CH_2Cl_2 (3 x 5 mL) then DMF (3 x 5 mL). Following the second coupling cycle of Fmoc-Ser(*t*Bu)-OH the resin was treated with 20 %(v/v) piperidine in DMF (2 x 10 min; 5 mL). The resin was drained and washed with DMF (3 x 5 mL), CH_2Cl_2 (3 x 5 mL) and DMF (3 x 5 mL). To the peptide resin was added a solution of Fmoc-Cys((*R*)-2,3-

bis(palmitoyloxy)propyl)-OH (3 equiv, 392 mg, 0.438 mmol), PyBOP (3 equiv, 228 mg, 0.438 mmol) and NMM (6 equiv, 0.096 mL, 0.876 mmol) in DMF (2 mL). The reaction was agitated for 45 min at room temperature then drained and washed with DMF (3 x 5 mL), CH_2Cl_2 (3 x 5 mL) and DMF (3 x 5 mL). A solution of 20 %(v/v) piperidine in DMF (5 mL) was added to the resin for 2 x 10 min. The resin was washed with DMF (3 x 5 mL), CH_2Cl_2 (3 x 5 mL), DMF (3 x 5 mL) and CH_2Cl_2 (3 x 5 mL). The resin was then dried under reduced pressure. The dry resin was swollen in CH_2Cl_2 (5 mL) under agitation for 20 min, then drained. The cleavage cocktail consisting of 95 %(v/v) TFA, 2.5% (v/v) TES and 2.5 %(v/v) H_2O (5 mL) was added to the syringe and agitated for 90 min. The cleavage cocktail was drained and collected. The resin was washed with cleavage cocktail (2 x 2.5 mL) and the combined solution was concentrated under a stream of N_2 followed by precipitation of the peptide with Et_2O (10 mL) at 0°C. The crude peptide was collected by centrifugation and washed with Et_2O (2 x 10 mL) at 0°C. The crude material was dried *in vacuo* at 0°C and the peptide was purified by silica column chromatography (CH_2Cl_2 – CH_2Cl_2:MeOH 9:1), and dried *in vacuo* at 0°C to yield FP2 as a white solid (14.0 mg, 9%).

TLC (CH_2Cl_2:MeOH 9:1) R_f = 0.31; ^{1}H NMR (600 MHz, DMSO-d_6) δ 8.21 – 8.13 (m, 3H, Lys-εNH, Cys-Ser-NH, Lys-Ser-NH), 7.87 (d, *J* = 7.9 Hz, 1H, Lys-NH), 7.45 (d, *J* = 8.0, Hz, 1H, Abz-Ar-CH), 7.16 – 7.07 (m, 3H, $CONH_2$, Abz-Ar-CH), 6.68 (d, *J* = 8.0 Hz, 1H, Abz-Ar-CH), 6.53 – 6.47 (m, 1H, Abz-Ar-CH), 6.35 (s, 2H, Abz-NH_2), 5.24 – 5.19 (m, 1H, Cys-Ser-OH), 5.14 – 5.08 (m, 1H, S-glyceryl-CH), 5.08 – 5.04 (m, 1H, Lys-Ser-OH), 4.41 - 4.36 (m, 1H, Cys-Ser-αCH), 4.34 – 4.26 (m, 2H, Lys-Ser-αCH, S-glyceryl-OCH_aH_b), 4.15 – 4.07 (m, 2H, Lys-αCH, S-glyceryl-OCH_aH_b), 3.72 – 3.64 (m, 2H, Cys-Ser-CH_aH_b, Lys-Ser-CH_aH_b), 3.61 – 3.55 (m, 1H, Lys-Ser-CH_aH_b), 3.55 – 3.49 (m, 1H, Cys-Ser-CH_aH_b), 3.41 – 3.38 (m, 1H, Cys-αCH), 3.17 (dd, *J* = 12.0, 5.6 Hz, 2H, Lys-εCH_2), 2.88 (dd, *J* = 13.3, 4.3

Hz, 1H, Cys-C$\underline{H_a}$$H_b$), 2.80 (dd, *J* = 14.1, 5.7 Hz, 1H, S-glyceryl-C$\underline{H_a}$$H_b$), 2.70 (dd, *J* = 14.1, 7.3 Hz, 1H, S-glyceryl-C$H_a$$\underline{H_b}$), 2.61 – 2.57 (m, 1H, Cys-C$H_a$$\underline{H_b}$), 2.31 – 2.24 (m, 4H, Pal-αCH_2 x 2), 1.78 – 1.71 (m, 1H, Lys-βC$\underline{H_a}$$H_b$), 1.57 – 1.42 (m, 7H, Lys-βC$H_a$$\underline{H_b}$, Pal-$CH_2$ x 2, Lys-*δ*CH_2), 1.38 – 1.32 (m, 2H, Lys-γCH_2), 1.31 – 1.17 (m, 48H, Pal-CH_2 x 24), 0.86 (t, *J* = 6.9 Hz, 6H, Pal-CH_3 x 2) ppm; ^{13}C NMR (151 MHz, DMSO-*d_6*) δ 173.8 ($CONH_2$), 173.4 (Cys C=O), 172.5 (Pal C=O), 172.3 (Pal C=O), 170.5 (Cys-<u>Ser</u> C=O), 169.7 (Lys-<u>Ser</u> C=O), 168.8 (Abz C=O), 149.5 (Abz qC-NH_2), 131.4 (Abz-Ar-CH), 128.0 (Abz-Ar-CH), 116.3 (Abz-Ar-CH), 115.0 (Abz qC), 114.5 (Abz Ar-CH), 70.0 (S-glyceryl-CH), 63.5 (S-glyceryl-OCH_2), 62.0 (Cys-<u>Ser</u>-βCH_2), 61.4 (Lys-<u>Ser</u>-βCH_2), 55.5 (Lys-<u>Ser</u>-αCH), 54.4 (Cys-<u>Ser</u>-αCH), 54.2 (Cys-αCH), 52.8 (Lys-αCH), 38.7 (Lys-εCH_2), 37.5 (Cys-βCH_2), 33.6 (Pal-αCH_2), 33.4 (Pal-αCH_2), 31.7 (Lys-βCH_2), 31.3 (S-glyceryl-CH_2), 29.1 , 29.0, 28.9, 28.8, 28.7, 28.6, 28.4, 28.4 (Pal-CH_2), 24.5 , 24.4 (Pal-βCH_2), 23.1 (Lys-γCH_2), 22.1 (Pal-CH_2), 13.9 (Pal-CH_3) ppm; *m/z* HRMS (ESI^+) calcd. $C_{57}H_{102}N_7O_{11}S$ = 1092.7353 $(M + H)^+$. Found = 1092.7350; ν_{max} (thin film)/cm^{-1}: 3287 (Amide A: NH), 2919 (CH_2), 2851 (CH_2), 1674 (Amide I: CO, CN), 1534 (Amide II: CN, NH), 1440, 1303 (Amide III: CN, CO), 1203 (CO).

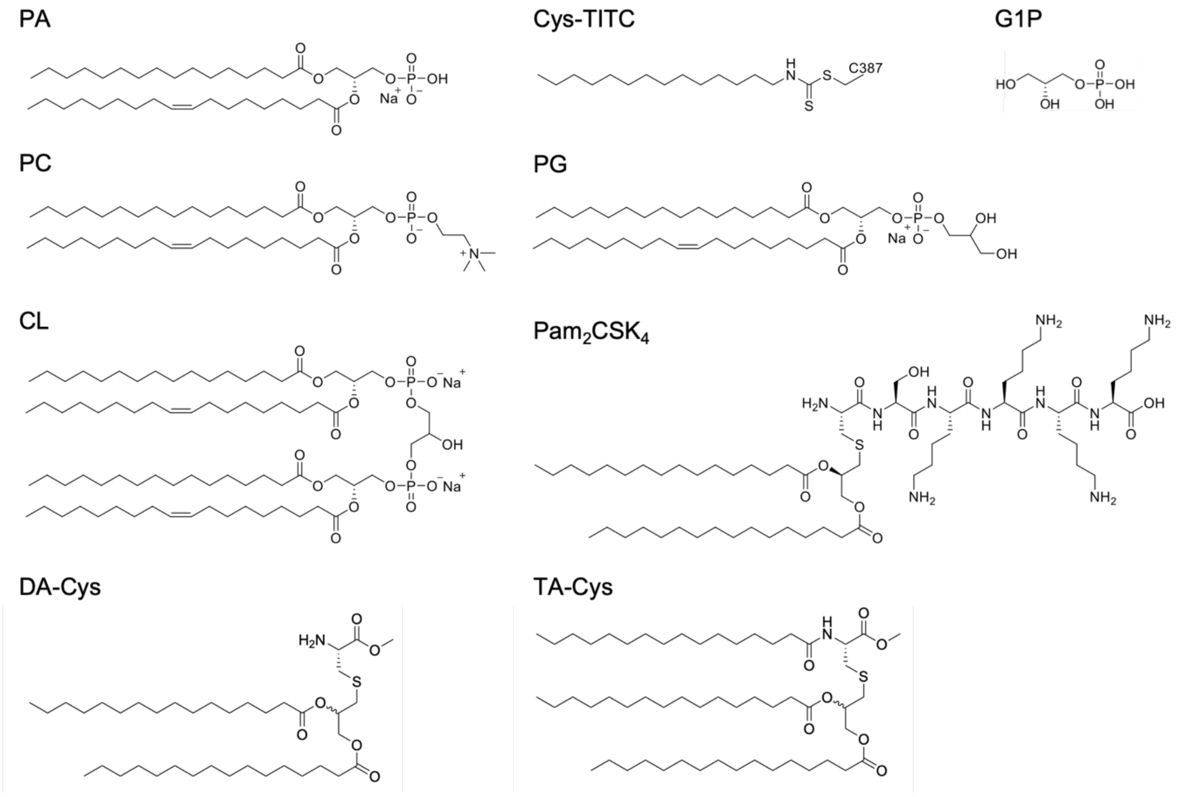

Fig. S1. Chemical structures of Lnt substrates, products and modification. PA = palmitoyl-oleoyl phosphatidic acid, PC = palmitoyl-oleoyl phosphatidylcholine, CL = palmitoyl-oleoyl cardiolipin, PG = palmitoyl-oleoyl phosphatidylglycerol, Cys-TITC = TITC-modified cysteine (C387 is the catalytic cysteine in Lnt*Eco*), G1P = glycerol-1-phosphate, DA-Cys = dipalmitoylglyceryl cysteine, TA-Cys = *N*-palmitoyl-dipalmitoylglyceryl cysteine.

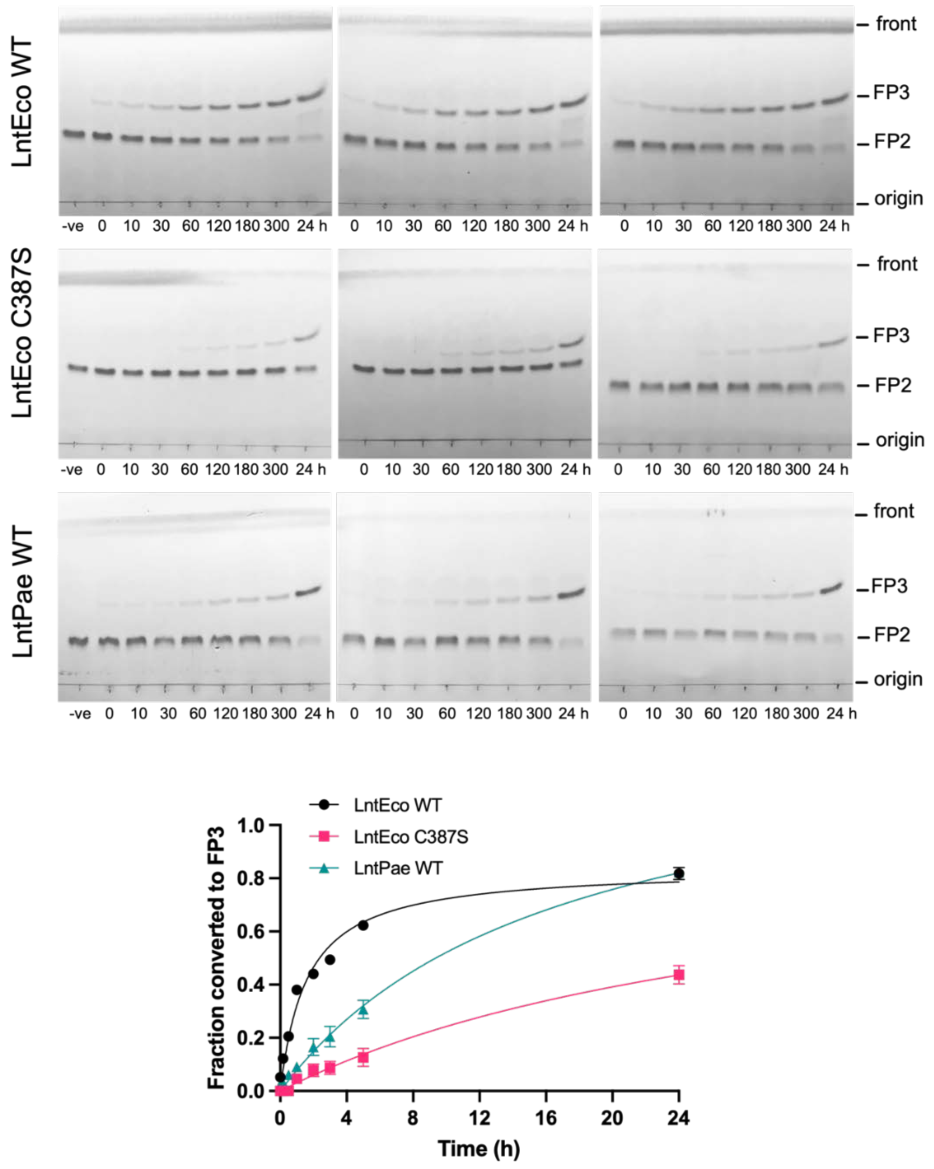

Fig. S2. Time-course assay of Lnt*Eco* WT, Lnt*Eco* C387S and Lnt*Pae* WT. Assays tracking the end-point conversion of FP2 to FP3 over time were run in triplicate using POPE as the GPL substrate. Each row represents one enzyme as indicated on the left. -ve = no enzyme control, taken after 24 h incubation at 37°C. Numbers below lanes correspond to the elapsed time in minutes, with the exception of the last time point which is at 24 h. Positions on the chromatogram of the front, FP3, FP2, and the origin are indicated to the right of each row. Densitometry analysis results obtained using ImageJ (*65*) are plotted in the lower panel as averages and standard deviations (n = 3). Best fit lines are provided to guide the eye.

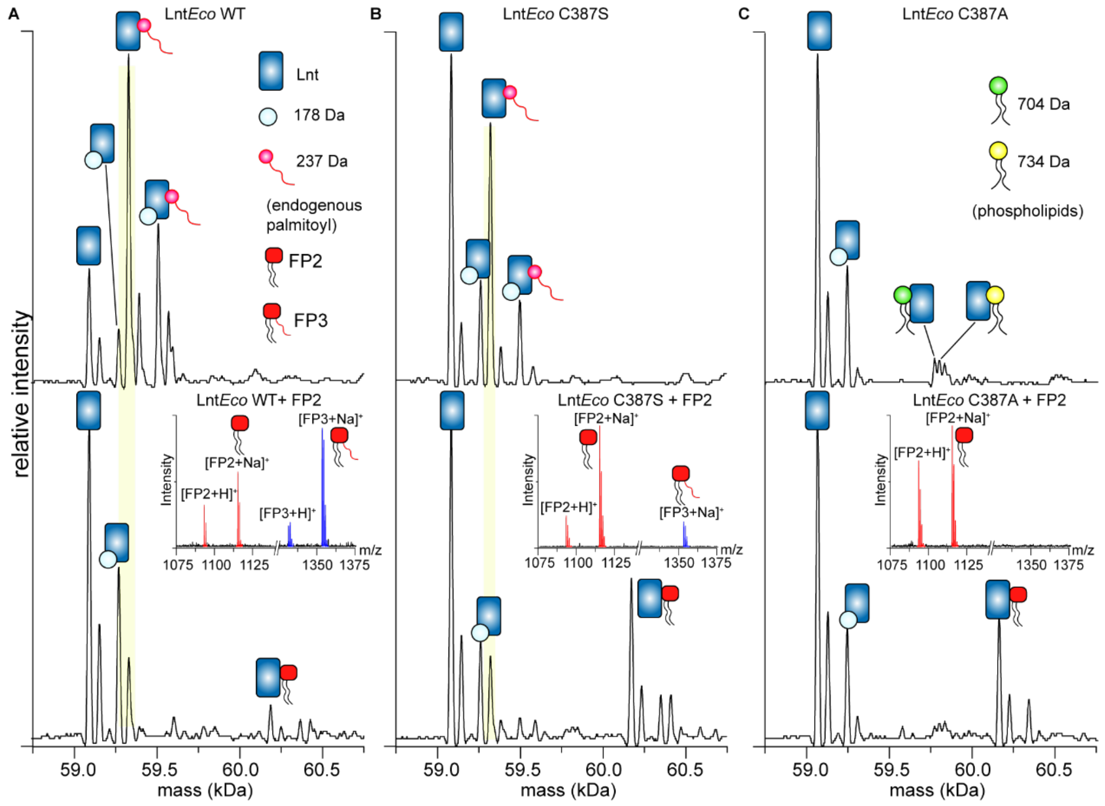

Fig. S3. Mass spectrometry analysis of Lnt*Eco* WT, C387S and C387A in the presence and absence of FP2. (**A**) Native mass spectra (deconvoluted) of purified Lnt*Eco* WT, (**B**) Lnt*Eco* C387S, and (**C**) Lnt*Eco* C387A in the absence of (top) and in the presence of FP2 (bottom). The WT and mutant variants of Lnt were incubated with FP2 for 1 h at 22 °C prior to making end-point measurements. Proteins were released from 200 mM ammonium acetate (pH 7.0) supplemented with 0.5 %(w/v) C8E4. All of the protein variants form adducts with a divalent cation (60 Da) and are covalently modified by α-N-6-phosphogluconoylation (178 Da). Unlike Lnt*Eco* C387A, the wild type and the C387S mutants are additionally palmitoylated (highlighted on a yellow background) and can transfer the palmitoyl group to FP2 (red rectangle). Inserts: spectra recorded for the Lnt-FP2 mixtures by tuning the instrument to enhance transmission of low m/z ions. Peaks corresponding to FP2 and FP3 ions are highlighted in red and blue, respectively.

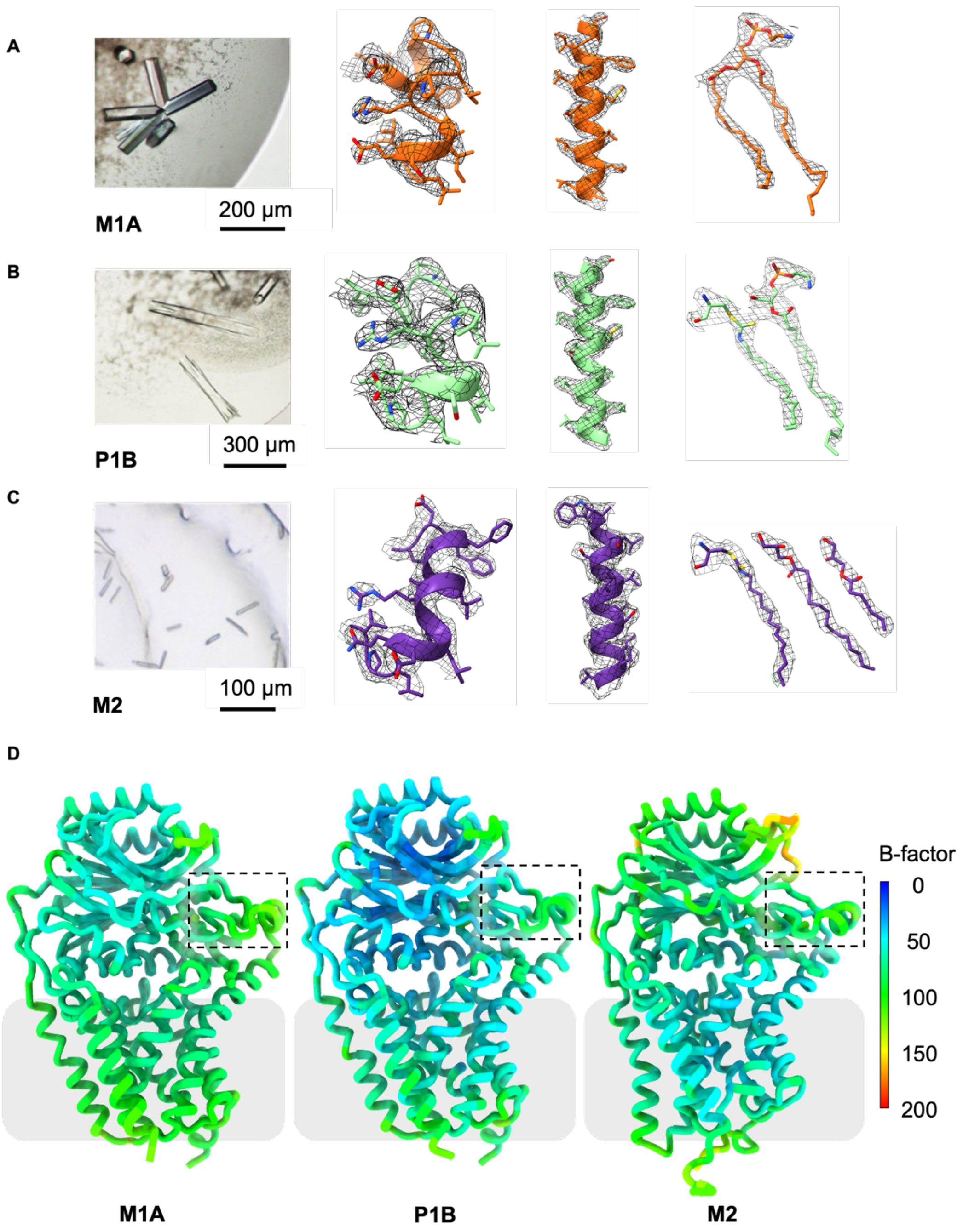

Fig. S4. Structures of Lnt solved by MX. (**A**) M1A MX structure (Lnt*Eco*-C387A-PE). From left to right; crystal image with scale bar, Arm 3 residues 345-360 in density, TM helix 6 residues 170-188 in density, and PE substrate in density. 2Fo-Fc maps represented in grey mesh contoured at 1.5 sigma, shown at a distance of 1.6 Å from atoms. Also included are Fo-Fc maps represented in green mesh, contoured at 3 sigma, shown at 1.6 Å from atoms. (**B**) P1B structure (Lnt*Eco*-TITC-LPE). Other details as in (**A**). Ligands include TITC and LPE. (**C**) M2 structure (Lnt*Pae*-TITC-MO). Other details as in (**A**). This is *Pae* orthologue where

the corresponding residues in Arm 3 are 340-355 and the residues for TM helix 6 are 168-183. Ligands include TITC-modified Cys382 and two monoolein molecules. 2Fo-Fc maps are contoured at 1 sigma, 1.6 Å from atoms shown in grey mesh. Fo-Fc maps are contoured at 3 sigma, 1.6 Å from atoms shown. (**D**) Structures of Lnt M1A, Lnt P1B and Lnt M2 colour coded by B-factor. A B-factor ($Å^2$) scale bar is provided to the right. Arm 3 is indicated by a dashed box on each structure.

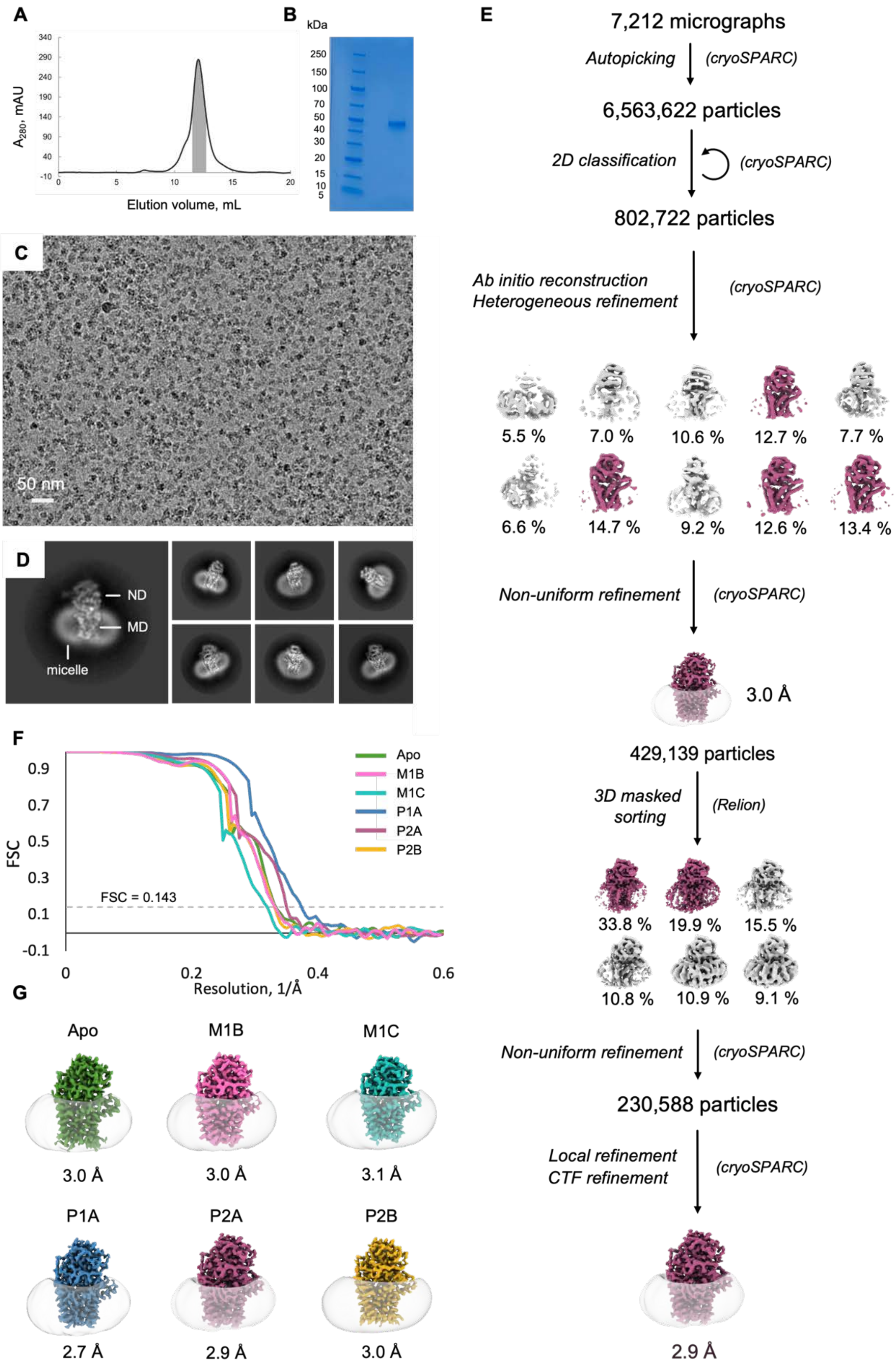

Fig. S5. Lnt purification and cryo-EM processing. (**A**) Size exclusion chromatography (SEC) profile of Lnt purified in LMNG. Fractions corresponding to the main peak of monomers (grey) were combined and used for the cryo-EM studies. (**B**) Coomassie-stained

SDS-PAGE showing purified Lnt protein after SEC. (**C**) An exemplary cryo-EM micrograph from the P2A complex structure sample. (**D**) Representative reference-free 2D class averages from the P2A complex structure sample in LMNG detergent micelles. ND = nitrilase domain, MD = membrane domain. (**E**) Processing flow chart of P2A complex cryo-EM data, including particle selection, 2D and 3D classifications, particle sorting, masking and final map reconstruction. Data processing was performed using a combination of CryoSPARC v 3.2 (*42*) and Relion v 3.1 (*66*). Software used for distinct steps are indicated in parenthesis within the diagram. Other cryo-EM data reported in this study were similarly processed, and the specific details are provided in Table S2. (**F**) Gold standard Fourier shell correlation (FSC) curves of half maps that were individually calculated indicating an average resolution at 0.143 FSC. (**G**) Final 3D cryo-EM maps of Lnt structures and their corresponding average resolution.

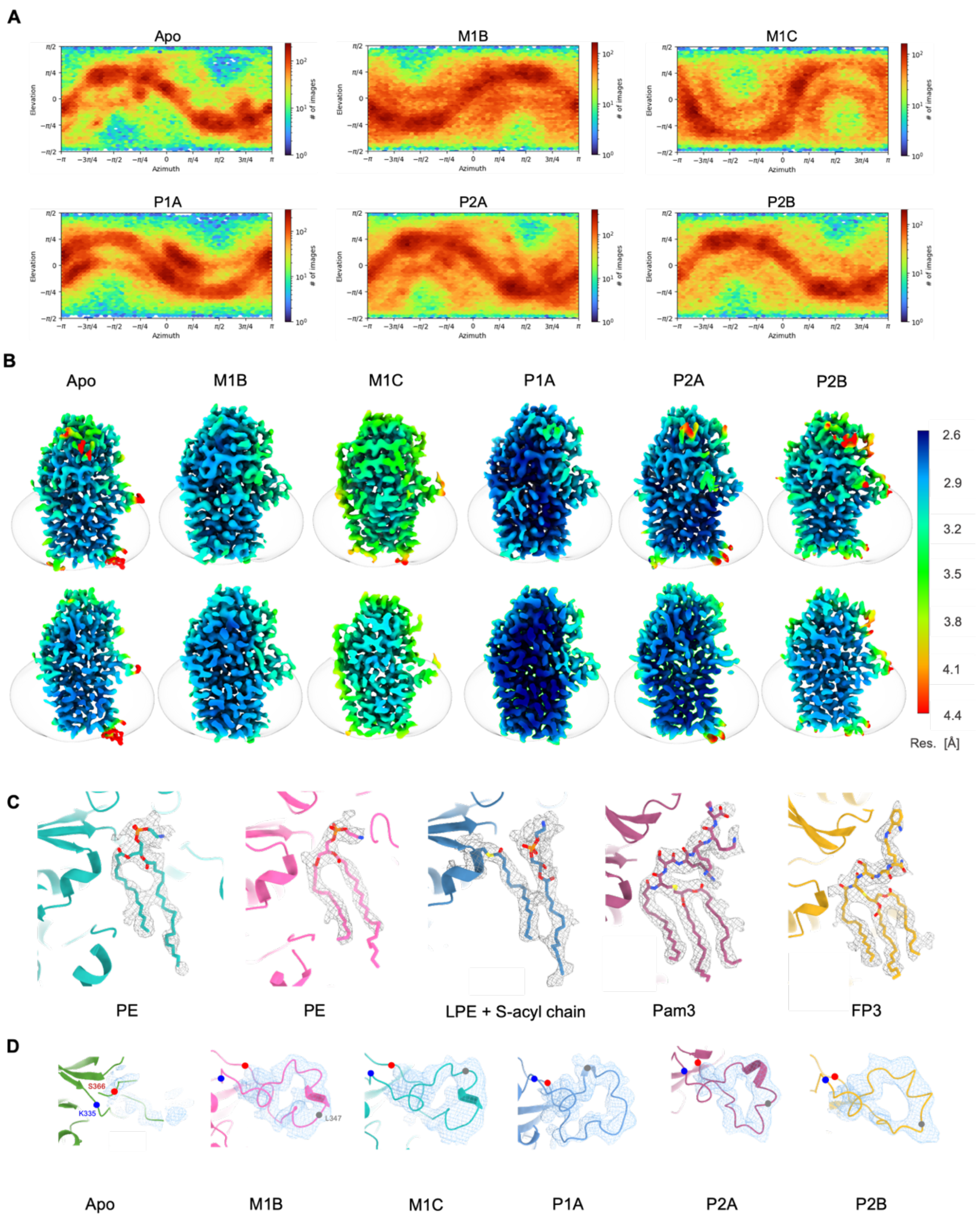

Fig. S6. Lnt map and model quality. (A) Angular distribution for particle projections. Heat maps show the number of particles for each viewing angle and were plotted using CryoSPARC v3.2 (*42*) **(B)** Final cryo-EM maps coloured by local resolution. Colour scheme for resolution in Å is presented to the right. Resolution distribution was calculated by CryoSPARC v3.2 (*42*) and plotted in ChimeraX v1.3 (*44*). (**C**) Lnt substrates, products and

modification in density. The ligands are represented as sticks and coloured according to the respective structures in **Fig. S5G**. **(D)** Lnt Arm 3 in density. The map shown is the unsharpened map and is contoured at 0.1 sigma level. The structure colours correspond to the colour scheme in **S5G**. Fiducials are provided to assist the reader in the form of a blue sphere (Lys335), grey sphere (Leu347) and red sphere (S366).

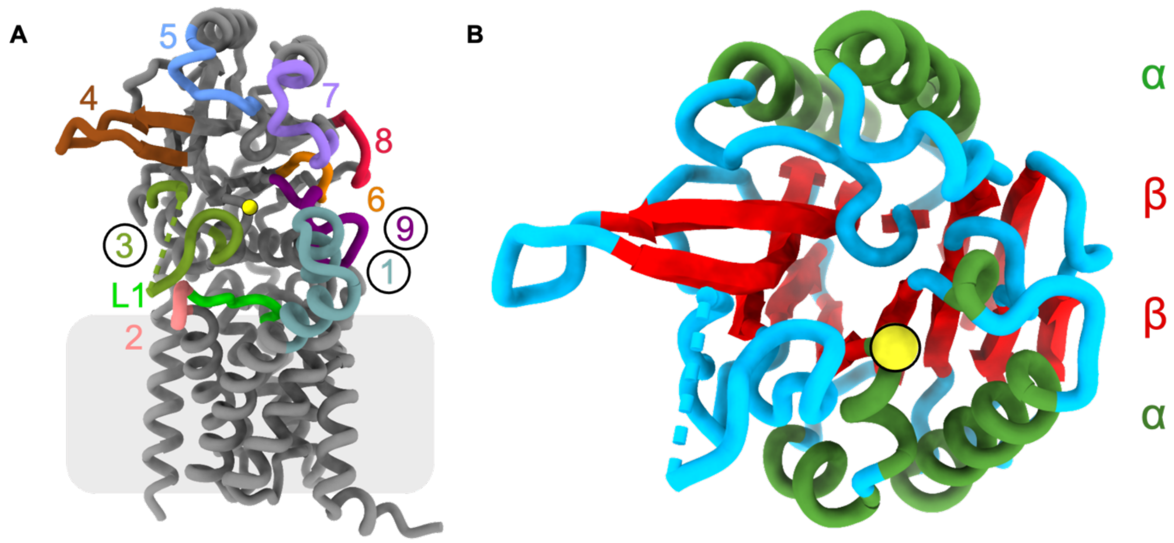

Fig. S7. Structural elements of Lnt*Eco*. (**A**) Identification of Arms 1-9 and Linker 1 (L1). Each arm and L1 is coloured differently and labelled. For orientation purposes, the catalytic Cys387 is shown as a yellow sphere with a black outline, and arms 1, 3, and 9, which frame the catalytic centre, are circled. (**B**) Identification of the αββα fold in the nitrilase-like domain. a-Helices, β-strands, and loops are coloured green, red, and blue, respectively.

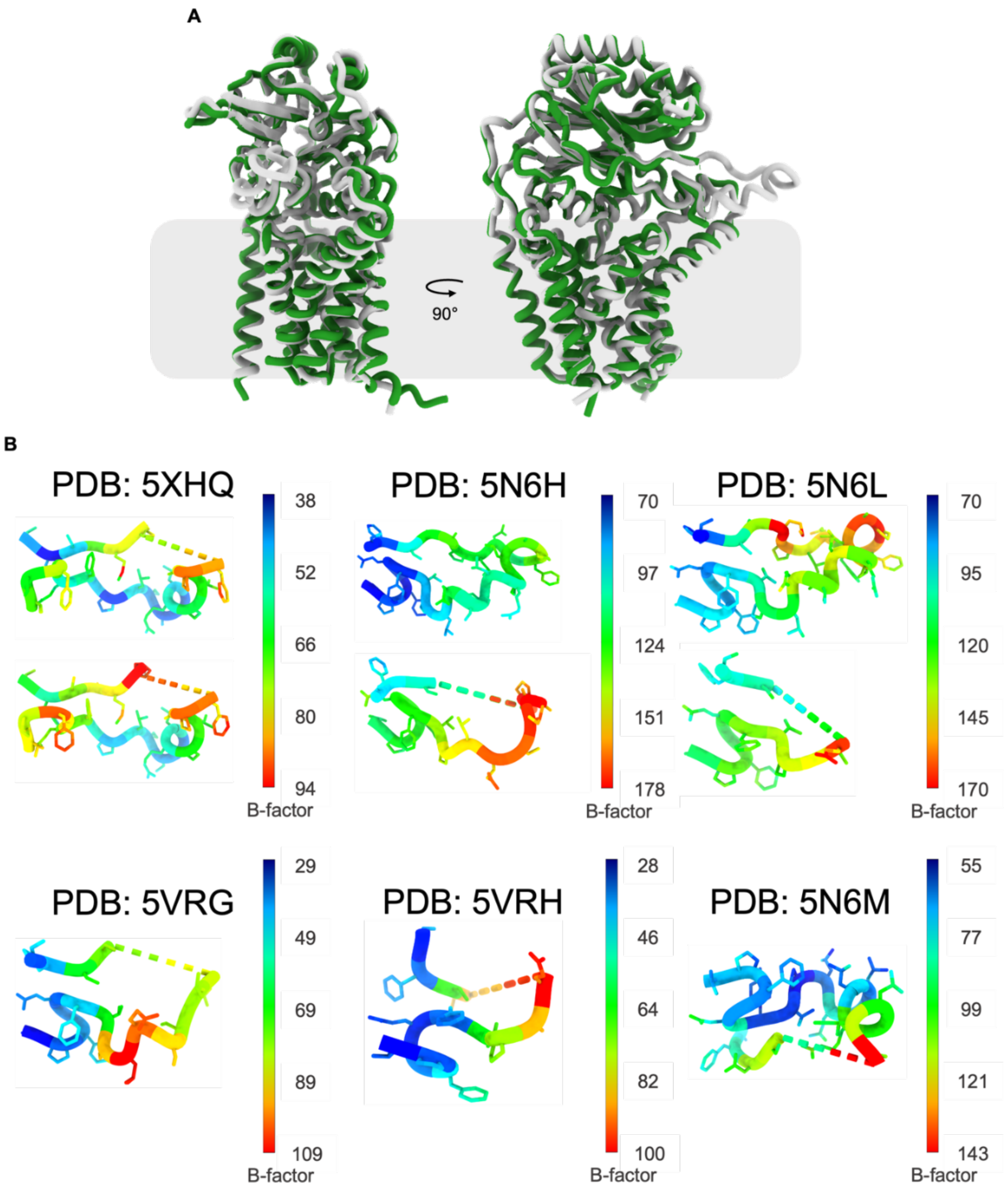

Fig. S8. Comparison of the cryo-EM apo structure with published MX structures of Lnt. (**A**) Alignment of cryo-EM apo structure (green) with MX structure in PDB record 5N6H (silver) (RMSD, 0.92 Å). (**B**) B-factor of Arm 3 for published apo structures of Lnt identified by PDB code. The B-factor is an indicator of thermal motion and disorder. The higher the B-factor value the more flexible is that part of the protein likely to be. Where there are two chains in the asymmetric unit, chain A is shown above and chain B below. Dashed lines correspond to regions that are not in density. A B-factor (Å^2) scale bar is provided to the right of each model.

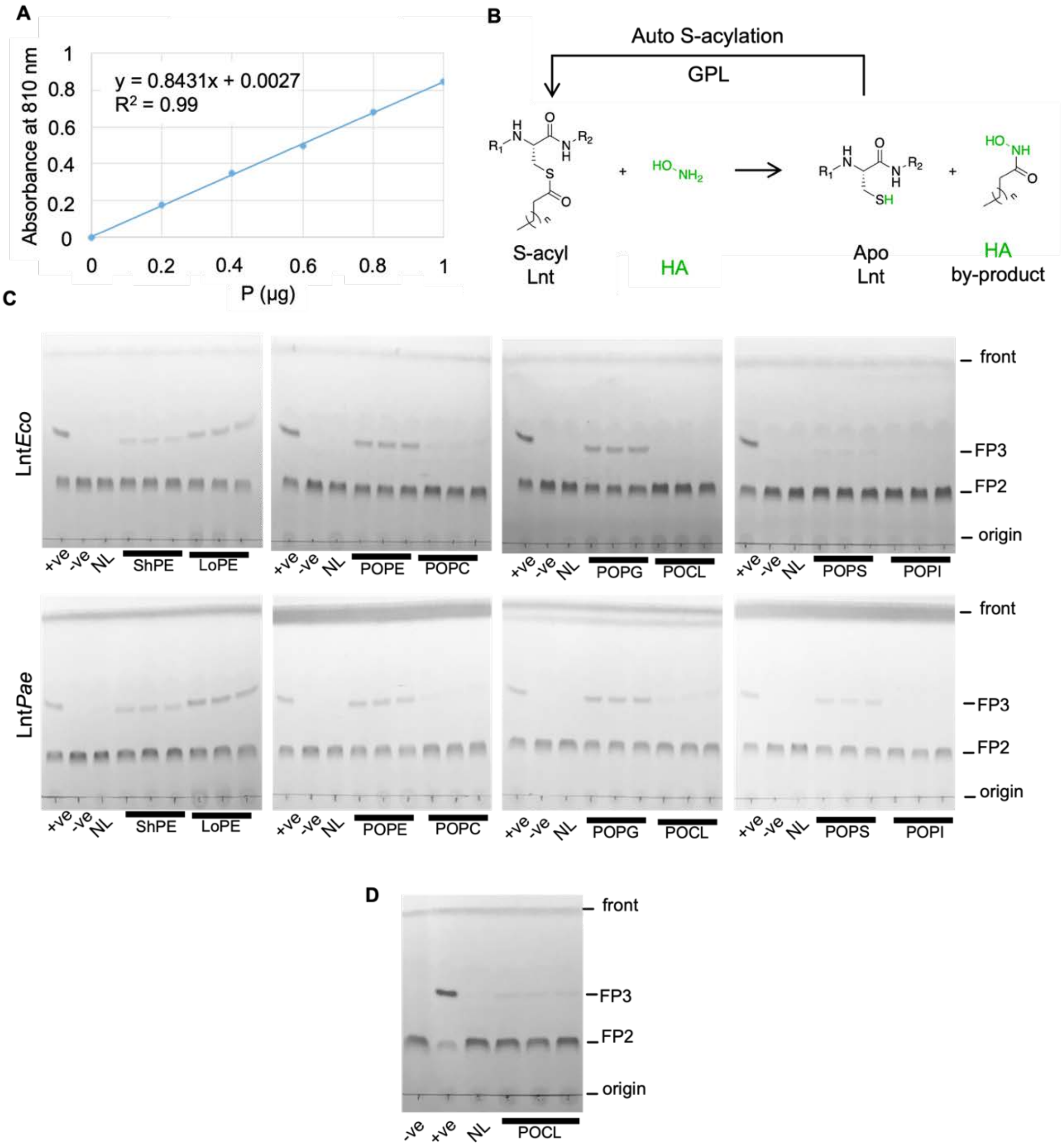

Fig. S9. Lnt co-purified phospholipid quantitation, hydroxylamine treatment and uncropped GPL specificity assays. (**A**) Standard curve for the phosphorous (P) determination assay used to quantify the amount of GPL co-purified with Lnt*Eco* WT. Duplicate data points are plotted along with the equation and correlation coefficient (R^2) for the line of best fit. For the analysis, duplicate Lnt*Eco* WT samples were used with an average mass of 80 mg protein corresponding to 1.4 nmol Lnt (MW of Lnt is 57 kDa). The average P content was 0.86 mg corresponding to 17.03 mg or 28.4 nmoles of GPLs (atomic mass of P is

31 Da; molecular mass of a GPL was estimated at 600 Da). Thus, (28.8/1.4 =) ~21 GPL molecules are bound per Lnt molecule. (**B**) Schematic describing how HA treatment deacylates the catalytic cysteine of Lnt and how co-purified GPL reacylates the enzyme. The latter undergoes repeated deacylation in the presence of excess HA until all GPL has been depleted. Reaction with HA produces the apo enzyme plus an acylated HA by-product. (**C**) Uncropped TLCs for the GPL specificity assay presented in Fig. 2G. (**D**) FP2 assay after 24 h with Lnt*Eco* and POCL as lipid substrate. A faint FP3 band is seen at the end of the incubation period (6.7 ±0.63 % conversion of FP2 to FP3).

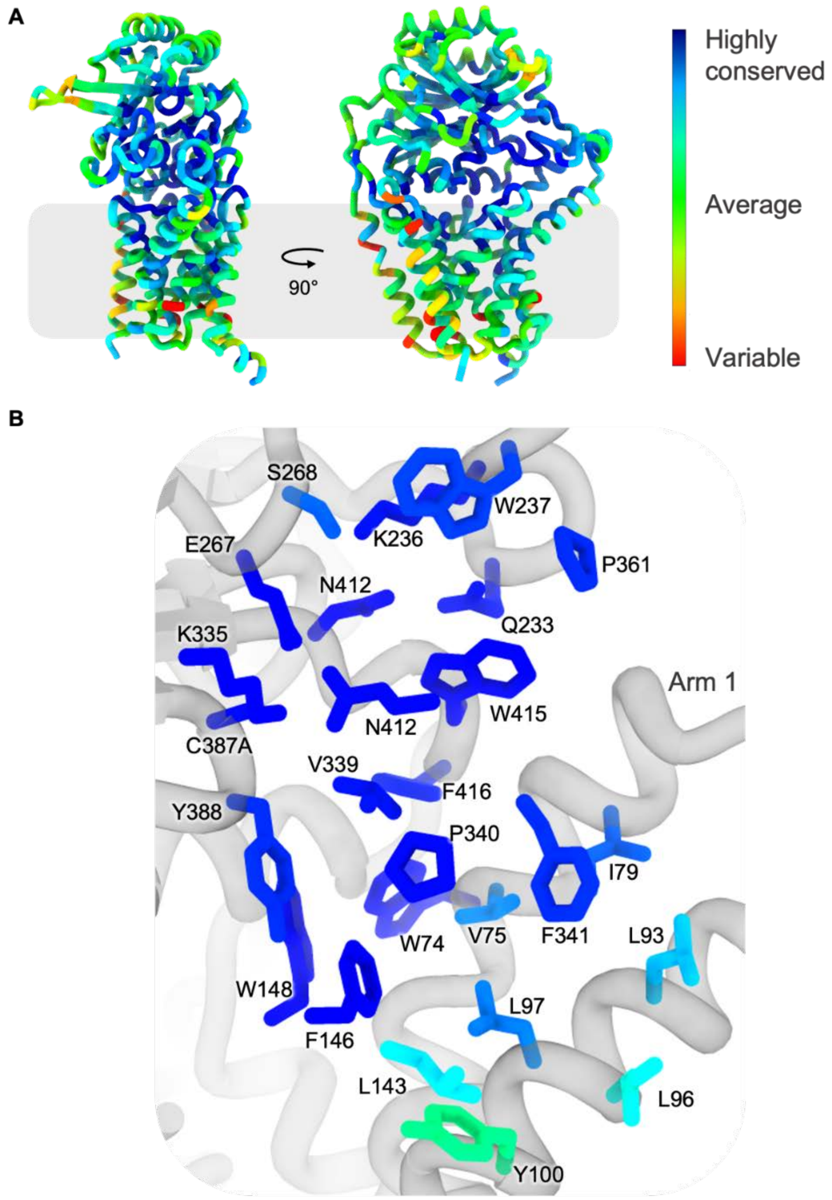

Fig. S10. ConSurf analysis of Lnt*Eco*. (**A**) Conserved residue surface representation of Lnt*Eco* (M1A) based on an analysis of 150 homologues with 35 to 95 % sequence identity with *E. coli* (strain K-12) Lnt using the ConSurf server (*67*) Lnt*Eco* in surface representation is coloured by conservation value in ChimeraX (*44*). Conservation values are reported in the Supplementary Materials (Database S1). (**B**) View into the active site with all residues identified as interacting with the substrates, acyl modification, and products, shown as sticks. Colouring is by conservation with the same scale bar as in (A).

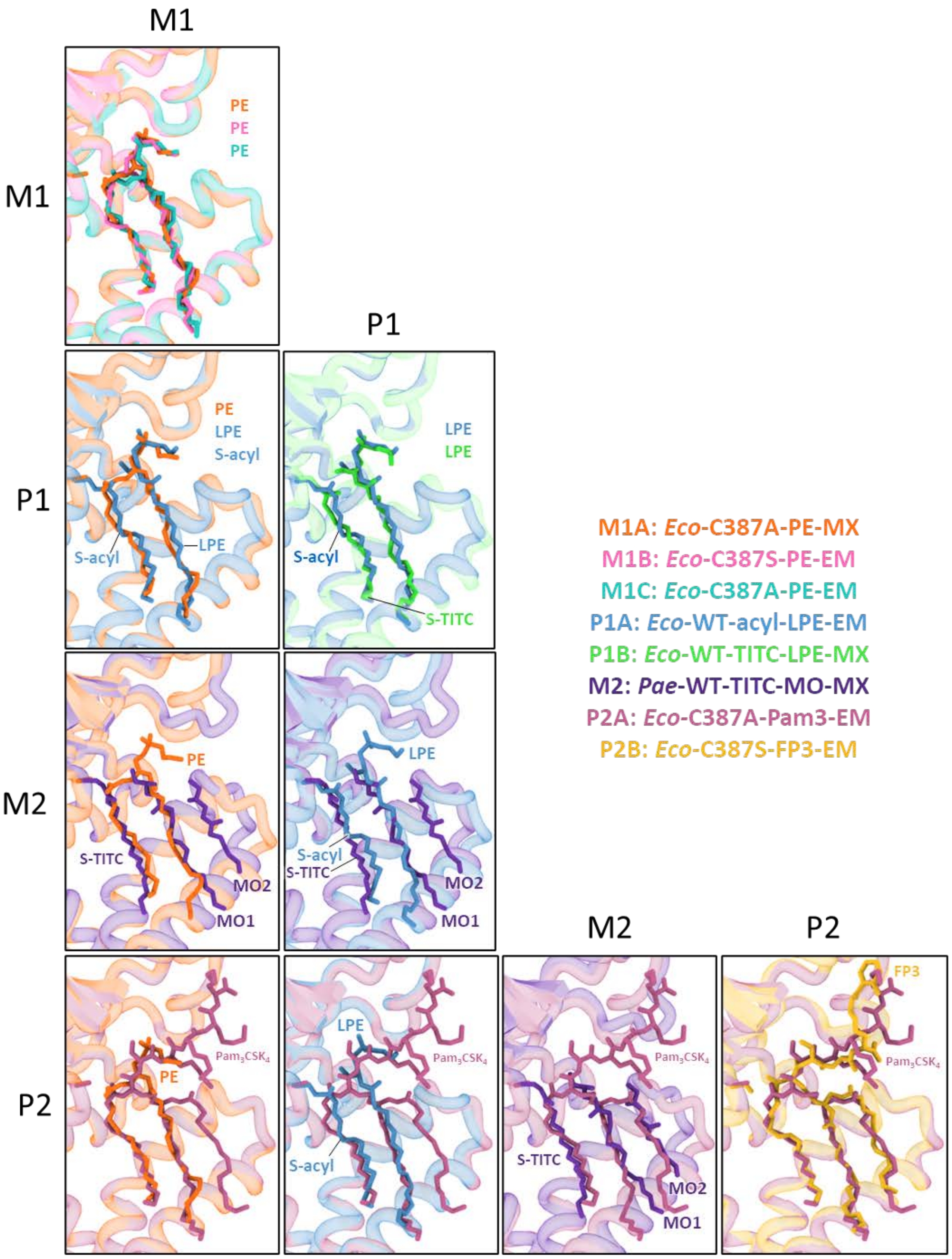

Fig. S11. Alignment matrix on the catalytic site of Lnt. A matrix across the eight 'ligand bound' structures solved (apo excluded) as part of this study is shown. Where the same structures intersect (as in M1 x M1, P1 x P1, and P2 x P2), all of the structures observed in that category are aligned. The colouring scheme follows that used in Fig. 1C and a colour-coded legend is provided to the right.

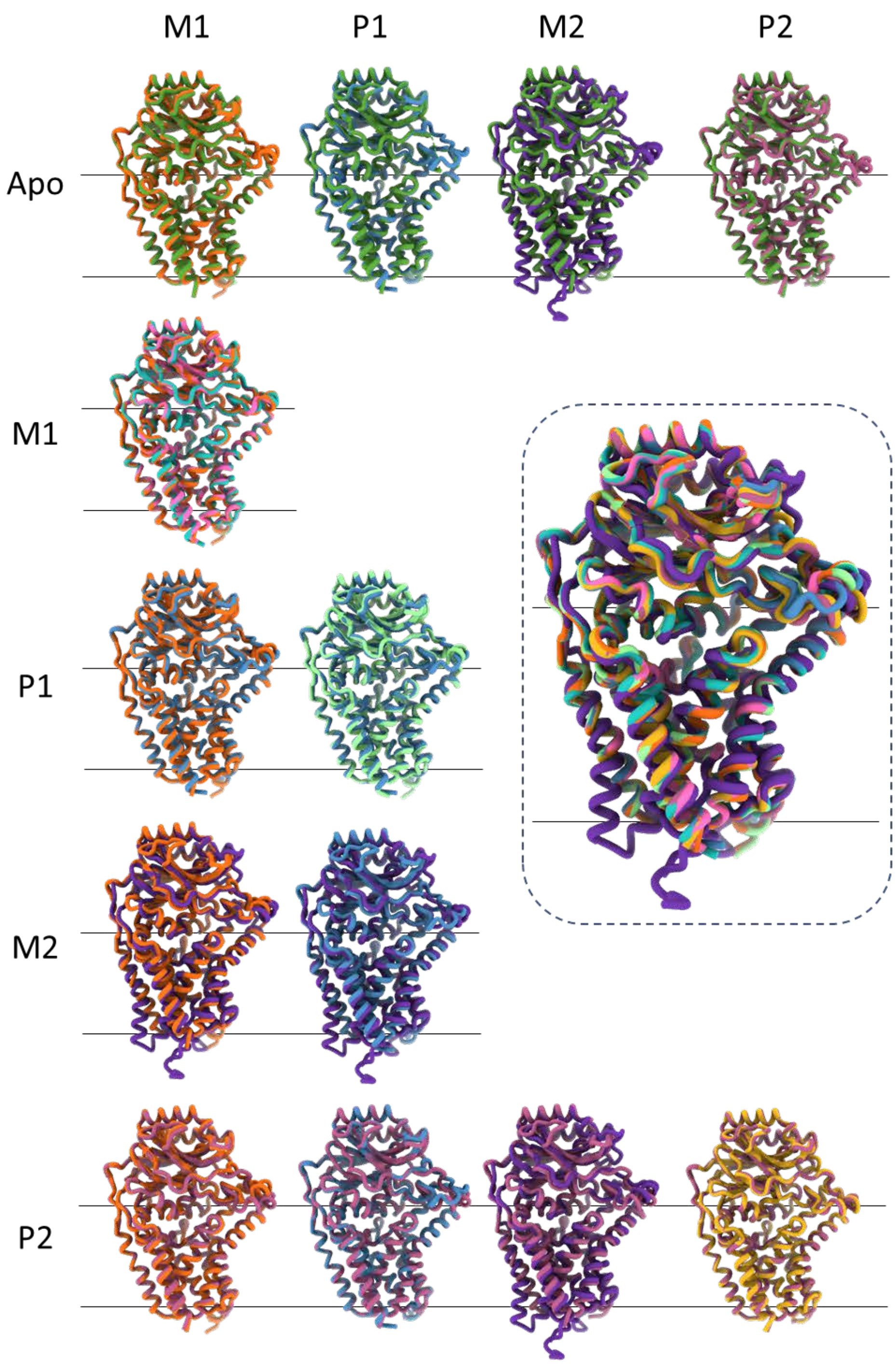

Fig. S12. Structure alignment matrix of the different states observed for Lnt in this study. Where the same structures intersect (as in M1 x M1, P1 x P1, and P2 x P2), all of the structures observed within that category are aligned. The colouring scheme follows that used in Fig. 1C. An alignment of all nine structures is shown in the dashed blue box inset.

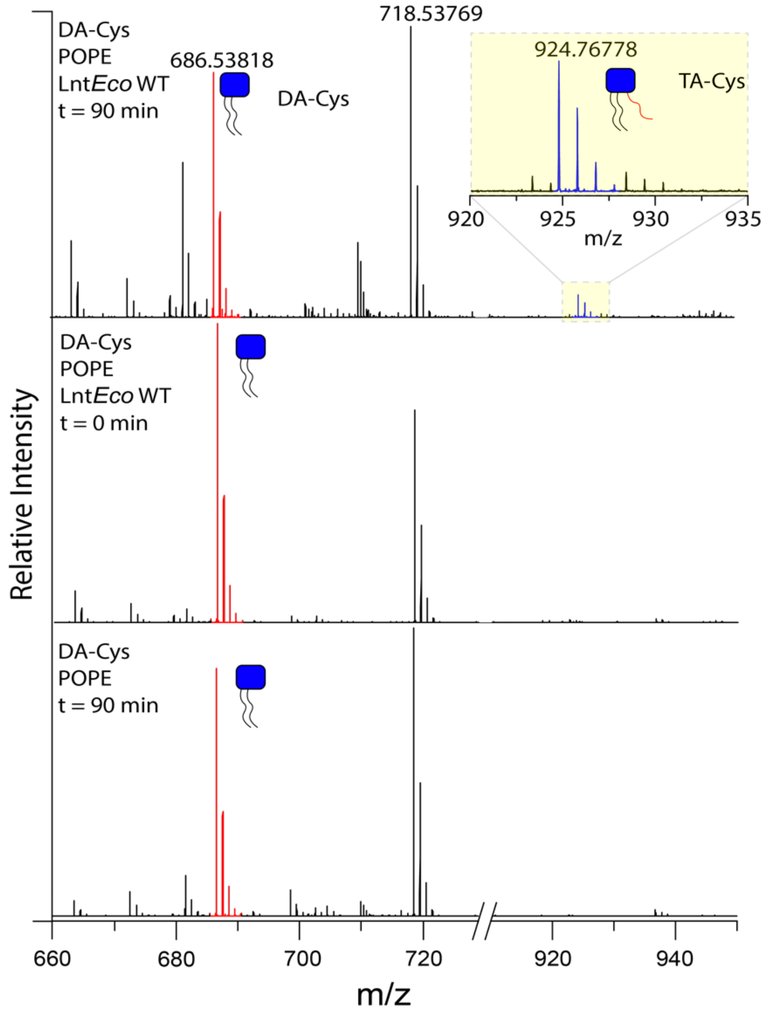

Fig. S13. Tracking the conversion of DA-Cys to TA-Cys by Lnt*Eco* using mass spectrometry. Lnt uses dipalmitoylglyceryl-cysteine (DA-Cys) as an acyl-accepting substrate. Shown are the spectra from reaction mixtures without Lnt (bottom), with Lnt at time 0 min (middle) and at 90 min (top). End-point measurements apply in all cases. TA-Cys is formed after 90 min incubation with POPE and Lnt (yellow inset top)

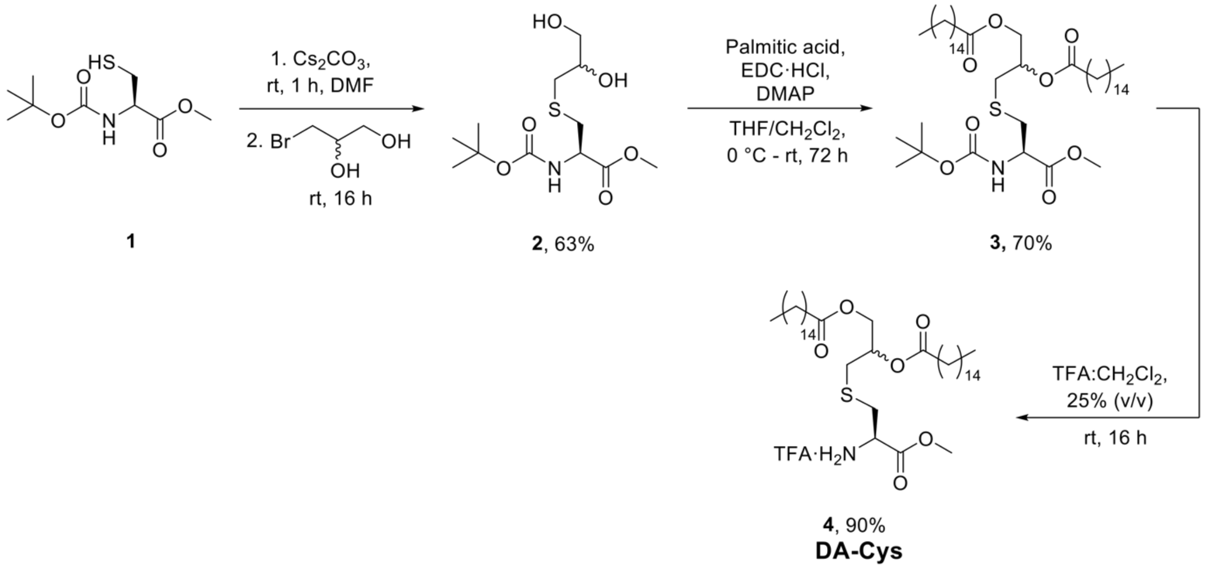

Fig. S14. Schematic for the synthesis of di-palmitoyl cysteine (DA-Cys)

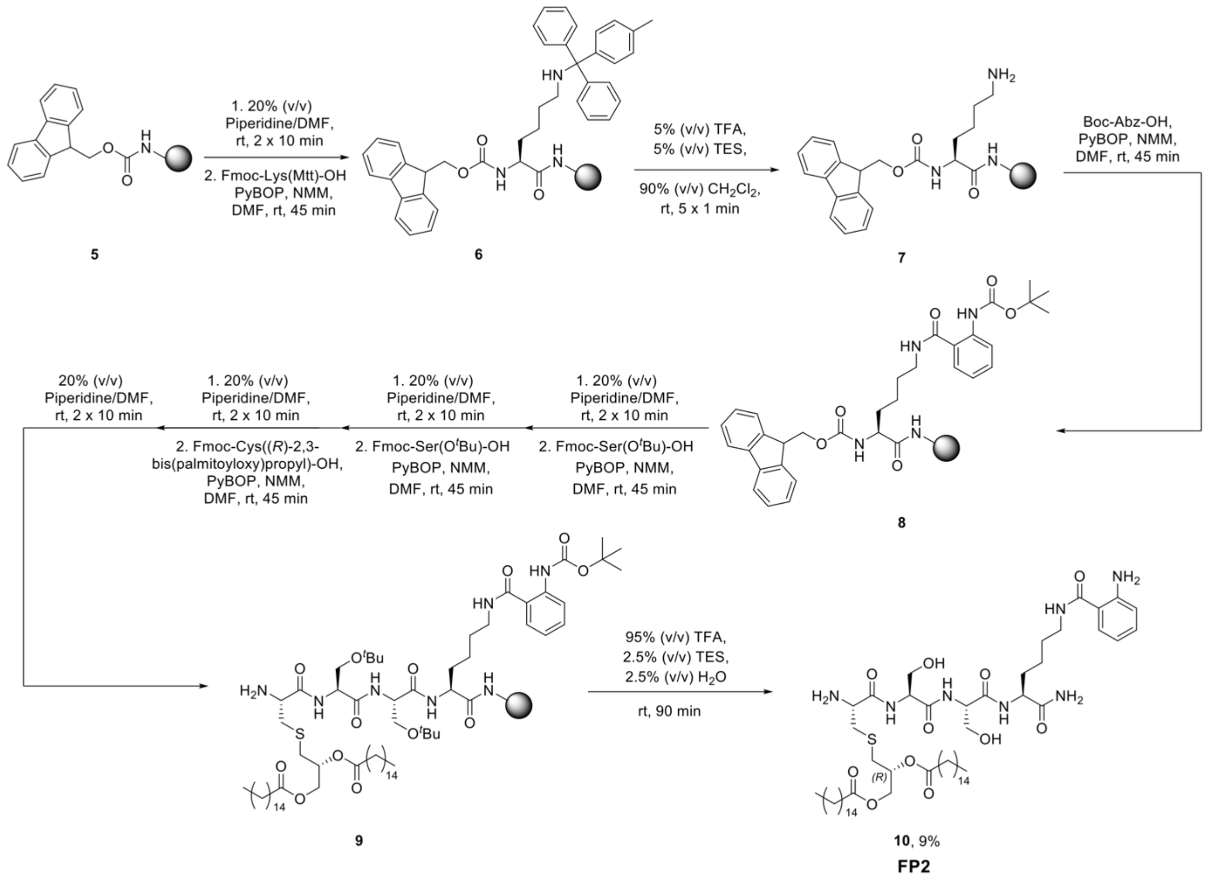

Fig. S15. Schematic for the solid phase peptide synthesis of FP2

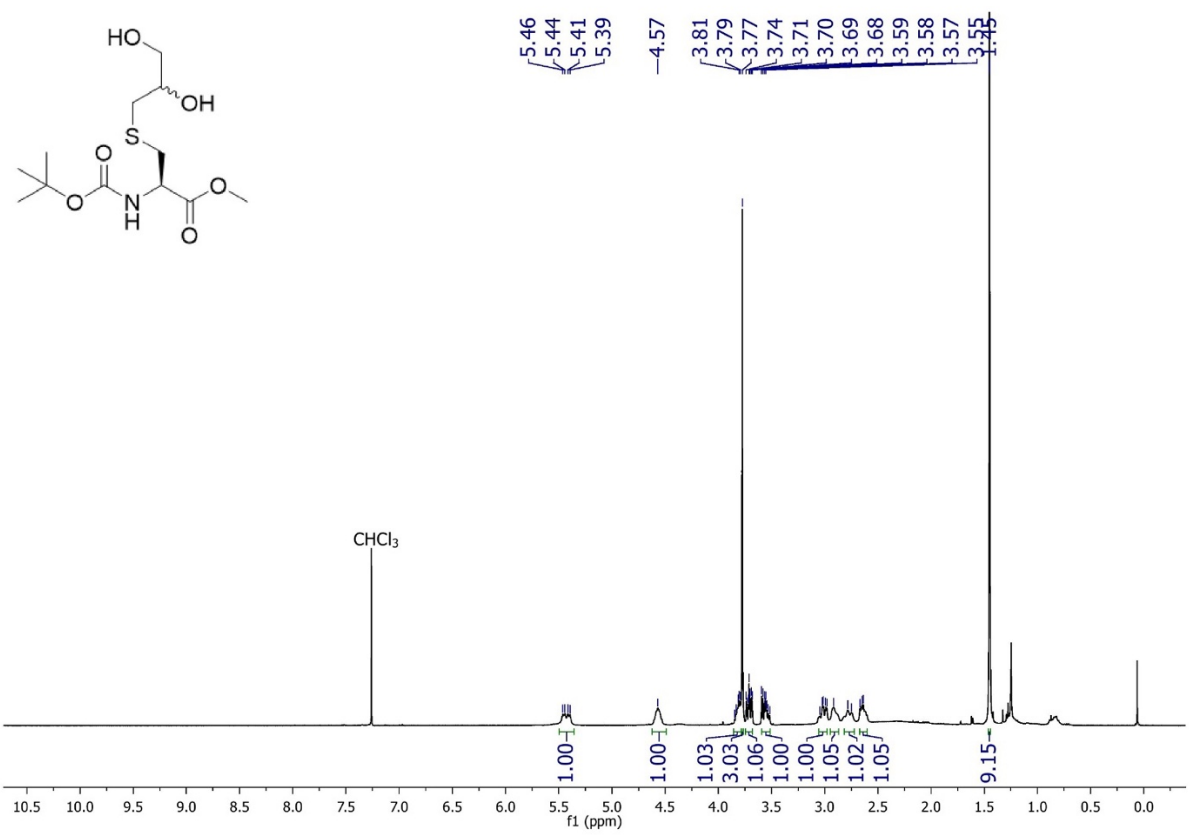

Fig. S16. ^{1}H NMR (400 MHz, $CDCl_3$) of the novel compound 2

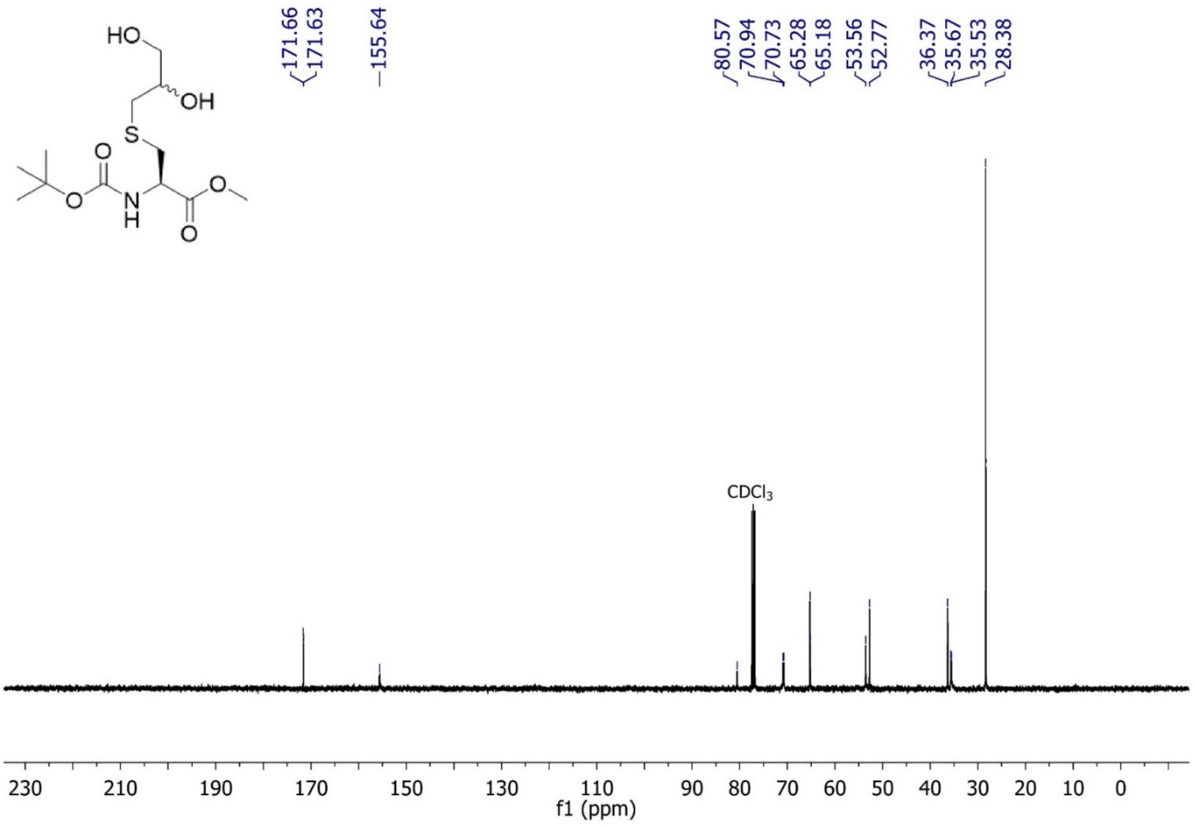

Fig. S17. **^{13}C NMR (101 MHz, $CDCl_3$) of the novel compound 2**

Table S1. (separate file) Macromolecular X-ray crystallographic data collection, refinement, and validation statistics

See TableS1.xlsx supplementary material excel file

Table S2. (separate file) Cryo-EM data collection, refinement, and validation statistics

See TableS2.xlsx supplementary material excel file

Table S3. Primers used for mutagenesis

**Primer name**	**Sequence ***
**Cys387Ala_Eco_Forward**	gaactgaccgcggcgatt<u>gcg</u>tatgaaattatcctggg
**Cys387Ala_Eco_Reverse**	cccaggataatttcata<u>cgc</u>aatcgccgcggtcagttc
**Cys387Ser_Eco_Forward**	gaactgaccgcggcgatt<u>agc</u>tatgaaattatcctggg
**Cys387Ser_Eco_Reverse**	cccaggataatttcata<u>gct</u>aatcgccgcggtcagttc

*Codons for mutated residues are underlined

Movie S1. (separate file) In surfo crystals of Lnt*Eco* C387A

Crystals of Lnt*Eco* C387A at 10 mg/mL in 20 mM Tris-HCl pH 8.0, 250 mM NaCl, 1 mM TCEP, 5 %(v/v) glycerol and 0.02 %(w/v) DDM were grown for 14 days at 18°C in a 24-well XRL plate by the hanging drop vapor diffusion method using 1 µL each of reservoir and protein solutions on 22 mm siliconised cover slips. Before crystallisation trials were set up the protein (25 µL) was incubated with *E. coli* polar lipid extract (300 µg) for 16 h at 4°C followed by centrifugation, to remove aggregates, at 20,000 x *g* for 90 min at 4°C. The reservoir solution (1 mL) consisted of 50 mM sodium acetate pH 5.0, 50 mM magnesium acetate, and 28–36 %(v/v) PEG 200 (*34*). The microscope used was an Olympus BX53 with a 20 x magnification lens (Olympus XLPlan N 25X/1.05W, water dipping) using a 455 - 490 nm emission filter. The fluorophores in Lnt were excited through a three-photon excitation at 750 nm (equivalent to one photon at 250 nm) using a multi photon Coherent Chameleon Laser.

Movie S2. (separate file) An X-ray/EM movie of the bacterial lipoprotein *N*-acyltransferase reaction

The ping-pong mechanism of the Lnt-catalysed lipoprotein *N*-acyltransfer reaction is shown in animated form. It is based on the 5 states and 9 structures captured by MX and cryo-EM during the course of the reaction. The structures shown are as close as possible to those recorded experimentally. However, for clarity, certain liberties were taken in their representation. For example, the M2 state is represented by the acylated enzyme complexed with DA-BLP obtained by molecular dynamics simulations (*37*) where, in fact, it was an M2 mimic (acyl-Lnt complexed with two monoolein molecules) that was recorded experimentally.

Database S1. (separate file) ConSurf data

Homologues were collected from UNIREF90 (*68*) using the HMMER search algorithm (*69, 70*) with an E-value cut-off of 0.0001 and default search parameters. 2,639 sequences passed the default thresholds for homologues. 150 unique hits were used for the multiple sequence alignment built using MAFFT (*71*). Conservation scores were then calculated in ConSurf (*67*) and mapped on to the M1A structure using ChimeraX (*44*).

REFERENCES AND NOTES

1. M. D. Resh, Covalent lipid modifications of proteins. *Curr. Biol.* **23**, R431–R435 (2013).

2. L. Smithers, S. Olatunji, M. Caffrey, Bacterial lipoprotein posttranslational modifications. New insights and opportunities for antibiotic and vaccine development. *Front. Microbiol.* **12**, 788445 (2021).

3. A. Kovacs-Simon, R. W. Titball, S. L. Michell, Lipoproteins of bacterial pathogens. *Infect. Immun.* **79**, 548–561 (2011).

4. W. G. Bessler, M. Cox, A. Lex, B. Suhr, K. H. Wiesmüller, G. Jung, Synthetic lipopeptide analogs of bacterial lipoprotein are potent polyclonal activators for murine B lymphocytes. *J. Immunol.* **135**, 1900–1905 (1985).

5. O. Takeuchi, T. Kawai, P. F. Mühlradt, M. Morr, J. D. Radolf, A. Zychlinsky, K. Takeda, S. Akira, Discrimination of bacterial lipoproteins by Toll-like receptor 6. *Int. Immunol.* **13**, 933–940 (2001).

6. O. Takeuchi, S. Sato, T. Horiuchi, K. Hoshino, K. Takeda, Z. Dong, R. L. Modlin, S. Akira, Cutting edge: Role of Toll-like receptor 1 in mediating immune response to microbial lipoproteins. *J. Immunol.* **169**, 10–14 (2002).

7. P. Xia, Y. Wu, S. Lian, L. Yan, X. Meng, Q. Duan, G. Zhu, Research progress on Toll-like receptor signal transduction and its roles in antimicrobial immune responses. *Appl. Microbiol. Biotechnol.* **105**, 5341–5355 (2021).

8. A. C. Steere, V. K. Sikand, F. Meurice, D. L. Parenti, E. Fikrig, R. T. Schoen, J. Nowakowski, C. H. Schmid, S. Laukamp, C. Buscarino, D. S. Krause, Vaccination against Lyme disease with recombinant *Borrelia burgdorferi* outer-surface lipoprotein A with adjuvant. Lyme Disease Vaccine Study Group. *N. Engl. J. Med.* **339**, 209–215 (1998).

9. E. Begier, D. J. Seiden, M. Patton, E. Zito, J. Severs, D. Cooper, J. Eiden, W. C. Gruber, K. U. Jansen, A. S. Anderson, A. Gurtman, SA4Ag, a 4-antigen *Staphylococcus aureus* vaccine, rapidly induces high levels of bacteria-killing antibodies. *Vaccine* **35**, 1132–1139 (2017).

10. N. J. Brendish, R. C. Read, *Neisseria meningitidis* serogroup B bivalent factor H binding protein vaccine. *Expert Rev. Vaccines* **14**, 493–503 (2015).

11. C. G. Brown, J. Clarke, Nanopore development at Oxford Nanopore. *Nat. Biotechnol.* **34**, 810–811 (2016).

12. A. Gandhi, P. Balmer, L. J. York, Characteristics of a new meningococcal serogroup B vaccine, bivalent rLP2086 (MenB-FHbp; Trumenba®). *Postgrad. Med.* **128**, 548–556 (2016).

13. Y. Luo, O. V. Friese, H. A. Runnels, L. Khandke, G. Zlotnick, A. Aulabaugh, T. Gore, E. Vidunas, S. W. Raso, E. Novikova, E. Byrne, M. Schlittler, D. Stano, R. L. Dufield, S. Kumar, A. S. Anderson, K. U. Jansen, J. C. Rouse, The dual role of lipids of the lipoproteins in Trumenba, a self-adjuvanting vaccine against meningococcal meningitis B disease. *AAPS J.* **18**, 1562–1575 (2016).

14. S. E. Van der Verren, N. Van Gerven, W. Jonckheere, R. Hambley, P. Singh, J. Kilgour, M. Jordan, E. J. Wallace, L. Jayasinghe, H. Remaut, A dual-constriction biological nanopore resolves homonucleotide sequences with high fidelity. *Nat. Biotechnol.* **38**, 1415–1420 (2020).

15. J. Diao, R. Komura, T. Sano, H. Pantua, K. M. Storek, H. Inaba, H. Ogawa, C. L. Noland, Y. Peng, S. L. Gloor, D. Yan, J. Kang, A. K. Katakam, M. Volny, P. Liu, N. N. Nickerson, W. Sandoval, C. D. Austin, J. Murray, S. T. Rutherford, M. Reichelt, Y. Xu, M. Xu, H. Yanagida, J. Nishikawa, P. C. Reid, C. N. Cunningham, S. B. Kapadia, Inhibition of *Escherichia coli* lipoprotein diacylglyceryl transferase is insensitive to resistance caused by deletion of Braun's lipoprotein. *J. Bacteriol.* **203**, e0014921 (2021).

16. K. Garland, H. Pantua, M.-G. Braun, D. J. Burdick, G. M. Castanedo, Y.-C. Chen, Y.-X. Cheng, J. Cheong, B. Daniels, G. Deshmukh, Y. Fu, P. Gibbons, S. L. Gloor, R. Hua, S. Labadie, X. Liu, R. Pastor, C. Stivala, M. Xu, Y. Xu, H. Zheng, S. B. Kapadia, E. J. Hanan, Optimization of globomycin analogs as novel Gram-negative antibiotics. *Bioorg. Med. Chem. Lett.* **30**, 127419 (2020).

17. T. Kiho, M. Nakayama, K. Yasuda, S. Miyakoshi, M. Inukai, H. Kogen, Synthesis and antimicrobial activity of novel globomycin analogues. *Bioorg. Med. Chem. Lett.* **13**, 2315–2318 (2003).

18. T. Kiho, M. Nakayama, K. Yasuda, S. Miyakoshi, M. Inukai, H. Kogen, Structure-activity relationships of globomycin analogues as antibiotics. *Bioorg. Med. Chem.* **12**, 337–361 (2004).

19. S. Kitamura, A. Owensby, D. Wall, D. W. Wolan, Lipoprotein signal peptidase inhibitors with antibiotic properties identified through design of a robust in vitro HT platform. *Cell Chem. Biol.* **25**, 301–308.e12 (2018).

20. S. Kitamura, D. W. Wolan, Probing substrate recognition of bacterial lipoprotein signal peptidase using FRET reporters. *FEBS Lett.* **592**, 2289–2296 (2018).

21. S. Olatunji, X. Yu, J. Bailey, C.Y. Huang, M. Zapotoczna, K. Bowen, M. Remškar, R. Müller, E. M. Scanlan, J. A. Geoghegan, V. Olieric, M. Caffrey, Structures of lipoprotein signal peptidase II from *Staphylococcus aureus* complexed with antibiotics globomycin and myxovirescin. *Nat. Commun.* **11**, 140 (2020).

22. H. Pantua, E. Skippington, M.-G. Braun, C. L. Noland, J. Diao, Y. Peng, S. L. Gloor, D. Yan, J. Kang, A. K. Katakam, J. Reeder, G. M. Castanedo, K. Garland, L. Komuves, M. Sagolla, C. D. Austin, J. Murray, Y. Xu, Z. Modrusan, M. Xu, E. J. Hanan, S. B. Kapadia, Unstable mechanisms of resistance to inhibitors of *Escherichia coli* lipoprotein signal peptidase. *MBio* **11**, e02018–e02020 (2020).

23. L. Vogeley, T. E. Arnaout, J. Bailey, P. J. Stansfeld, C. Boland, M. Caffrey, Structural basis of lipoprotein signal peptidase II action and inhibition by the antibiotic globomycin. *Science* **351**, 876–880 (2016).

24. K. J. Huang, H. Pantua, J. Diao, E. Skippington, M. Volny, W. Sandoval, V. Tiku, Y. Peng, M. Sagolla, D. Yan, J. Kang, A. K. Katakam, N. Michaelian, M. Reichelt, M.W. Tan, C. D. Austin, M. Xu, E. Hanan, S. B. Kapadia, Deletion of a previously uncharacterized lipoprotein lirL confers resistance to an inhibitor of type II signal peptidase in *Acinetobacter baumannii*. *Proc. Natl. Acad. Sci. U.S.A.* **119**, e2123117119 (2022).

25. M. S. Jin, S. E. Kim, J. Y. Heo, M. E. Lee, H. M. Kim, S.-G. Paik, H. Lee, J.-O. Lee, Crystal structure of the TLR1-TLR2 heterodimer induced by binding of a tri-acylated lipopeptide. *Cell* **130**, 1071–1082 (2007).

26. J. Y. Kang, X. Nan, M. S. Jin, S.-J. Youn, Y. H. Ryu, S. Mah, S. H. Han, H. Lee, S.-G. Paik, J.-O. Lee, Recognition of lipopeptide patterns by Toll-like receptor 2-Toll-like receptor 6 heterodimer. *Immunity* **31**, 873–884 (2009).

27. S. Sharma, R. Zhou, L. Wan, S. Feng, K. Song, C. Xu, Y. Li, M. Liao, Mechanism of LolCDE as a molecular extruder of bacterial triacylated lipoproteins. *Nat. Commun.* **12**, 4687 (2021).

28. S. Jackowski, C. O. Rock, Transfer of fatty acids from the 1-position of phosphatidylethanolamine to the major outer membrane lipoprotein of Escherichia coli. *J. Biol. Chem.* **261**, 11328–11333 (1986).

29. F. Hillmann, M. Argentini, N. Buddelmeijer, Kinetics and phospholipid specificity of apolipoprotein N-acyltransferase. *J. Biol. Chem.* **286**, 27936–27946 (2011).

30. N. Buddelmeijer, The molecular mechanism of bacterial lipoprotein modification—How, when and why? *FEMS Microbiol. Rev.* **39**, 246–261 (2015).

31. S. Gélis-Jeanvoine, S. Lory, J. Oberto, N. Buddelmeijer, Residues located on membrane-embedded flexible loops are essential for the second step of the apolipoprotein N-acyltransferase reaction. *Mol. Microbiol.* **95**, 692–705 (2015).

32. S. D. Gupta, W. Dowhan, H. C. Wu, Phosphatidylethanolamine is not essential for the N-acylation of apolipoprotein in Escherichia coli. *J. Biol. Chem.* **266**, 9983–9986 (1991).

33. S. D. Gupta, H. C. Wu, Identification and subcellular localization of apolipoprotein N-acyltransferase in Escherichia coli. *FEMS Microbiol. Lett.* **62**, 37–41 (1991).

34. G. Lu, Y. Xu, K. Zhang, Y. Xiong, H. Li, L. Cui, X. Wang, J. Lou, Y. Zhai, F. Sun, X. C. Zhang, Crystal structure of *E. coli* apolipoprotein N-acyl transferase. *Nat. Commun.* **8**, 15948 (2017).

35. C. L. Noland, M. D. Kattke, J. Diao, S. L. Gloor, H. Pantua, M. Reichelt, A. K. Katakam, D. Yan, J. Kang, I. Zilberleyb, M. Xu, S. B. Kapadia, J. M. Murray, Structural insights into lipoprotein N-acylation by *Escherichia coli* apolipoprotein N-acyltransferase. *Proc. Natl. Acad. Sci. U.S.A.* **114**, E6044-E6053 (2017).

36. L. van Dalsen, Smithers, L., Boland, C., Weichart, D., Caffrey, M., 9.8 MAG: A new host lipid for *In Meso* (lipid cubic phase) crystallization of integral membrane proteins. *Cryst. Growth Des.* **21**, 490–500 (2021).

37. M. Wiktor, D. Weichert, N. Howe, C.-Y. Huang, V. Olieric, C. Boland, J. Bailey, L. Vogeley, P. J. Stansfeld, N. Buddelmeijer, M. Wang, M. Caffrey, Structural insights into the mechanism of the membrane integral N-acyltransferase step in bacterial lipoprotein synthesis. *Nat. Commun.* **8**, 15952 (2017).

38. B. Wiseman, M. Högbom, Conformational changes in Apolipoprotein N-acyltransferase (Lnt). *Sci. Rep.* **10**, 639 (2020).

39. N. Buddelmeijer, R. Young, The essential *Escherichia coli* apolipoprotein N-acyltransferase (Lnt) exists as an extracytoplasmic thioester acyl-enzyme intermediate. *Biochemistry* **49**, 341–346 (2010).

40. M. M. Babu, M. L. Priya, A. T. Selvan, M. Madera, J. Gough, L. Aravind, K. Sankaran, A database of bacterial lipoproteins (DOLOP) with functional assignments to predicted lipoproteins. *J. Bacteriol.* **188**, 2761–2773 (2006).

41. M. T. Marty, A. J. Baldwin, E. G. Marklund, G. K. A. Hochberg, J. L. P. Benesch, C. V. Robinson, Bayesian deconvolution of mass and ion mobility spectra: From binary interactions to polydisperse ensembles. *Anal. Chem.* **87**, 4370–4376 (2015).

42. A. Punjani, J. L. Rubinstein, D. J. Fleet, M. A. Brubaker, cryoSPARC: Algorithms for rapid unsupervised cryo-EM structure determination. *Nat. Methods* **14**, 290–296 (2017).

43. J. Zivanov, T. Nakane, B. O. Forsberg, D. Kimanius, W. J. H. Hagen, E. Lindahl, S. H. W. Scheres, New tools for automated high-resolution cryo-EM structure determination in RELION-3. *eLife* **7**, e42166 (2018).

44. E. F. Pettersen, T. D. Goddard, C. C. Huang, E. C. Meng, G. S. Couch, T. I. Croll, J. H. Morris, T. E. Ferrin, UCSF ChimeraX: Structure visualization for researchers, educators, and developers. *Protein Sci.* **30**, 70–82 (2021).

45. O. S. W. Smart, T. O. Womack, A. Sharff, C. Flensburg, P. Keller, W. Paciorek, C. Vonrhein, G. Bricogne, Grade, version 1.2.20 (Global Phasing Ltd., 2011).

46. E. F. Pettersen, T. D. Goddard, C. C. Huang, G. S. Couch, D. M. Greenblatt, E. C. Meng, T. E. Ferrin, UCSF Chimera—A visualization system for exploratory research and analysis. *J. Comput. Chem.* **25**, 1605–1612 (2004).

47. P. Emsley, K. Cowtan, Coot: Model-building tools for molecular graphics. *Acta Crystallogr. D Biol. Crystallogr.* **60**, 2126–2132 (2004).

48. P. V. Afonine, B. K. Poon, R. J. Read, O. V. Sobolev, T. C. Terwilliger, A. Urzhumtsev, P. D. Adams, Real-space refinement in *PHENIX* for cryo-EM and crystallography. *Acta Crystallogr D Struct. Biol.* **74**, 531–544 (2018).

49. V. B. Chen, W. B. Arendall III, J. J. Headd, D. A. Keedy, R. M. Immormino, G. J. Kapral, L. W. Murray, J. S. Richardson, D. C. Richardson, MolProbity: All-atom structure validation for macromolecular crystallography. *Acta Crystallogr. D Biol. Crystallogr.* **66**, 12–21 (2010).

50. M. Caffrey, C. Porter, Crystallizing membrane proteins for structure determination using lipidic mesophases. e1712 (2010).

51. M. Caffrey, V. Cherezov, Crystallizing membrane proteins using lipidic mesophases. *Nat. Protoc.* **4**, 706–731 (2009).

52. A. Cheng, B. Hummel, H. Qiu, M. Caffrey, A simple mechanical mixer for small viscous lipid-containing samples. *Chem. Phys. Lipids* **95**, 11–21 (1998).

53. D. Li, C. Boland, K. Walsh, M. Caffrey, Use of a robot for high-throughput crystallization of membrane proteins in lipidic mesophases. *J. Vis. Exp.* e4000 (2012).

54. C. Y. Huang, V. Olieric, P. Ma, N. Howe, L. Vogeley, X. Liu, R. Warshamanage, T. Weinert, E. Panepucci, B. Kobilka, K. Diederichs, M. Wang, M. Caffrey, *In meso in situ* serial X-ray crystallography of soluble and membrane proteins at cryogenic temperatures. *Acta Crystallogr. D Struct. Biol.* **72**, 93–112 (2016).

55. J. A. Wojdyla, J. W. Kaminski, E. Panepucci, S. Ebner, X. Wang, J. Gabadinho, M. Wang, DA+ data acquisition and analysis software at the Swiss Light Source macromolecular crystallography beamlines. *J. Synchrotron Radiat.* **25**, 293–303 (2018).

56. J. A. Wojdyla, E. Panepucci, I. Martiel, S. Ebner, C.-Y. Huang, M. Caffrey, O. Bunk, M. Wang, Fast two-dimensional grid and transmission X-ray microscopy scanning methods for visualizing and characterizing protein crystals. *J. Appl. Crystallogr.* **49**, 944–952 (2016).

57. W. Kabsch, XDS. *Acta Crystallogr. D Biol. Crystallogr.* **66**, 125–132 (2010).

58. C. Vonrhein, C. Flensburg, P. Keller, A. Sharff, O. Smart, W. Paciorek, T. Womack, G. Bricogne, Data processing and analysis with the autoPROC toolbox. *Acta Crystallogr. D Biol. Crystallogr.* **67**, 293–302 (2011).

59. I. J. Tickle, C. Flensburg, P. Keller, W. Paciorek, A. Sharff, C. Vonrhein, G. Bricogne, STARANISO (Global Phasing Ltd., 2018).

60. A. J. McCoy, R. W. Grosse-Kunstleve, P. D. Adams, M. D. Winn, L. C. Storoni, R. J. Read, Phaser crystallographic software. *J. Appl. Crystallogr.* **40**, 658–674 (2007).

61. G. Bricogne, E. Blanc, M. Brandl, C. Flensburg, P. Keller, W. Paciorek, P. Roversi, A. Sharff, O. S. Smart, C. Vonrhein, T. O. Womack, BUSTER (Global Phasing Ltd., 2017).

62. D. Li, M. Caffrey, Renaturing membrane proteins in the lipid cubic phase, a nanoporous membrane mimetic. *Sci. Rep.* **4**, 5806 (2014).

63. W. Wu, R. Li, S. S. Malladi, H. J. Warshakoon, M. R. Kimbrell, M. W. Amolins, R. Ukani, A. Datta, S. A. David, Structure-activity relationships in toll-like receptor-2 agonistic diacylthioglycerol lipopeptides. *J. Med. Chem.* **53**, 3198–3213 (2010).

64. S. Olatunji, K. Bowen, C.-Y. Huang, D. Weichert, W. Singh, I. G. Tikhonova, E. M. Scanlan, V. Olieric, M. Caffrey, Structural basis of the membrane intramolecular transacylase reaction responsible for lyso-form lipoprotein synthesis. *Nat. Commun.* **12**, 4254 (2021).

65. C. A. Schneider, W. S. Rasband, K. W. Eliceiri, NIH Image to ImageJ: 25 years of image analysis. *Nat. Methods* **9**, 671–675 (2012).

66. S. H. W. Scheres, RELION: Implementation of a Bayesian approach to cryo-EM structure determination. *J. Struct. Biol.* **180**, 519–530 (2012).

67. H. Ashkenazy, S. Abadi, E. Martz, O. Chay, I. Mayrose, T. Pupko, N. Ben-Tal, ConSurf 2016: An improved methodology to estimate and visualize evolutionary conservation in macromolecules. *Nucleic Acids Res.* **44**, W344–W350 (2016).

68. B. E. Suzek, Y. Wang, H. Huang, P. B. McGarvey, C. H. Wu; the UniProt Consortium, UniRef clusters: A comprehensive and scalable alternative for improving sequence similarity searches. *Bioinformatics* **31**, 926–932 (2015).

69. R. D. Finn, J. Clements, S. R. Eddy, HMMER web server: Interactive sequence similarity searching. *Nucleic Acids Res.* **39**, W29–W37 (2011).

70. S. C. Potter, A. Luciani, S. R. Eddy, Y. Park, R. Lopez, R. D. Finn, HMMER web server: 2018 update. *Nucleic Acids Res.* **46**, W200-W204 (2018).

71. K. Katoh, J. Rozewicki, K. D. Yamada, MAFFT online service: Multiple sequence alignment, interactive sequence choice and visualization. *Brief. Bioinform.* **20**, 1160–1166 (2019).
